# Supplementary material for: Reliability of surface electromyographic (sEMG) measures of equine axial and appendicular muscles during overground trot
Source: PLoS One. 2023 Jul 14;18(7):e0288664. doi: 10.1371/journal.pone.0288664 (PMC10348569; doi:10.1371/journal.pone.0288664)
Supplement: S1 Table — (PDF) [file pone.0288664.s001.pdf]

S1 Table. Within- and between-day intra-subject coefficients of variation (CV) from each of the studied horses (n = 8), muscles and test days (day 1 and day 2).

		Biceps femoris		Superficial gluteal		Latissimus dorsi		Longissimus L1		Longissimus T14		Semitendinosus		Triceps brachii	
		Left	Right	Left	Right	Left	Right	Left	Right	Left	Right	Left	Right	Left	Right
Horse 1	Day 1	0.84	0.77	0.53	0.59	0.63	0.54	0.51	0.31	0.42	0.59	0.62	0.98	0.74	0.77
	Day 2	0.61	0.67	0.58	0.65	0.64	0.63	0.51	0.80	0.43	0.44	0.52	0.85	0.61	0.74
	Between-day	0.80	0.92	0.73	0.65	0.66	0.65	0.52	0.66	0.45	0.54	0.61	0.91	0.69	0.79
Horse 2	Day 1	0.71	0.78	0.59	0.63	0.47	0.50	0.46	0.41	0.37	0.62	0.82	0.91	0.63	0.73
	Day 2	0.47	1.12	0.56	0.42	0.47	0.65	0.43	0.44	0.46	0.49	0.39	0.56	0.87	1.15
	Between-day	0.75	1.12	0.69	0.42	0.48	0.56	0.55	0.44	0.50	0.69	0.84	0.87	0.85	0.86
Horse 3	Day 1	0.77	0.67	0.48	0.58	0.57	0.59	0.36	0.41	0.40	0.42	0.74	0.75	0.82	0.57
	Day 2	0.82	0.78	0.47	0.52	0.54	0.51	0.34	0.43	1.00	0.42	0.73	0.60	0.66	0.60
	Between-day	0.84	0.83	0.50	0.52	0.59	0.56	0.36	0.52	0.90	0.57	0.79	0.70	0.87	0.60
Horse 4	Day 1	0.74	0.53	0.54	0.53	0.47	0.45	0.37	0.41	0.33	0.40	0.54	0.63	0.73	0.66
	Day 2	0.76	0.99	0.52	0.53	0.52	0.47	0.37	0.39	0.40	0.41	0.65	0.64	0.73	0.68
	Between-day	0.92	0.86	0.56	0.53	0.54	0.49	0.39	0.49	0.44	0.41	0.75	0.66	0.77	0.70
Horse 5	Day 1	0.69	0.76	0.60	0.60	0.76	0.41	0.43	0.38	0.42	0.46	0.82	0.63	0.61	0.57
	Day 2	0.76	0.74	0.68	0.68	0.76	0.64	0.51	0.65	0.52	0.53	0.93	0.69	0.67	0.84
	Between-day	0.74	0.82	0.66	0.66	0.83	0.58	0.57	0.57	0.59	0.51	1.12	0.67	0.66	0.75
Horse 6	Day 1	0.64	0.66	0.52	-	0.52	0.40	0.40	0.45	0.57	0.48	0.53	-	0.75	0.59
	Day 2	0.53	1.11	0.58	0.72	0.54	0.49	0.45	0.57	0.52	0.50	0.60	0.68	0.66	0.89
	Between-day	0.61	1.04	0.57	-	0.54	0.47	0.44	0.51	0.72	0.49	0.67	-	0.71	0.75
Horse 7	Day 1	0.65	0.65	0.49	0.53	0.62	0.52	0.32	0.33	0.33	0.38	0.66	0.56	0.56	0.86
	Day 2	0.65	0.69	0.38	0.47	0.44	0.52	0.38	0.33	0.35	0.34	0.47	0.46	0.39	0.57
	Between-day	0.83	0.69	0.51	0.47	0.59	0.54	0.55	0.39	0.39	0.40	0.74	0.67	0.50	0.80
Horse 8	Day 1	0.51	0.55	0.53	0.50	0.59	0.70	0.45	0.63	0.54	0.82	0.63	0.58	0.74	0.48
	Day 2	0.74	0.52	0.55	0.50	0.47	0.48	0.29	0.26	0.66	0.46	0.65	0.77	0.41	0.40
	Between-day	0.76	0.60	0.56	0.50	0.64	0.82	0.46	0.57	0.69	0.75	0.74	0.70	0.88	0.58
