# Supplementary material for: Reliability of surface electromyographic (sEMG) measures of equine axial and appendicular muscles during overground trot
Source: PLoS One. 2023 Jul 14;18(7):e0288664. doi: 10.1371/journal.pone.0288664 (PMC10348569; doi:10.1371/journal.pone.0288664)
Supplement: S2 Table — (PDF) [file pone.0288664.s002.pdf]

S2 Table. Between-day, intra-subject coefficient of multiple correlation (CMC) from each of the studied horses (n = 8) and muscles, calculated across test days (day 1 and day 2).

		Horse 1	Horse 2	Horse 3	Horse 4	Horse 5	Horse 6	Horse 7	Horse 8
Biceps femoris	Left	0.81	0.32	0.84	-	0.97	0.62	0.97	0.97
	Right	0.98	0.99	0.97	1.00	0.97	0.99	0.95	0.90
Superficial gluteal	Left	0.93	0.98	0.94	0.98	0.97	0.91	0.99	0.99
	Right	0.86	0.86	0.83	0.94	0.90	-	0.96	0.94
Latissimus dorsi	Left	0.75	0.65	0.96	0.98	0.97	1.00	0.80	0.95
	Right	0.99	0.94	0.86	0.41	-	0.94	0.99	0.97
Longissimus L1	Left	0.77	0.95	0.94	0.95	0.97	0.98	0.82	-
	Right	0.97	0.92	0.69	0.98	0.97	0.98	0.99	0.97
Longissimus T14	Left	0.99	0.99	0.99	0.97	0.99	0.98	0.96	0.85
	Right	0.94	0.97	0.98	0.98	0.99	0.99	0.98	0.95
Semitendinosus	Left	0.81	1.00	0.99	0.99	0.90	0.99	0.97	1.00
	Right	0.93	0.88	0.95	1.00	0.99	-	0.98	0.98
Triceps brachii	Left	0.88	0.96	0.95	0.97	0.94	0.95	0.97	1.00
	Right	0.99	0.88	1.00	0.97	0.97	0.97	0.99	0.99
