# Supplementary figures and images for: Reliability of surface electromyographic (sEMG) measures of equine axial and appendicular muscles during overground trot
Source: PLoS One. 2023 Jul 14;18(7):e0288664. doi: 10.1371/journal.pone.0288664 (PMC10348569; doi:10.1371/journal.pone.0288664)

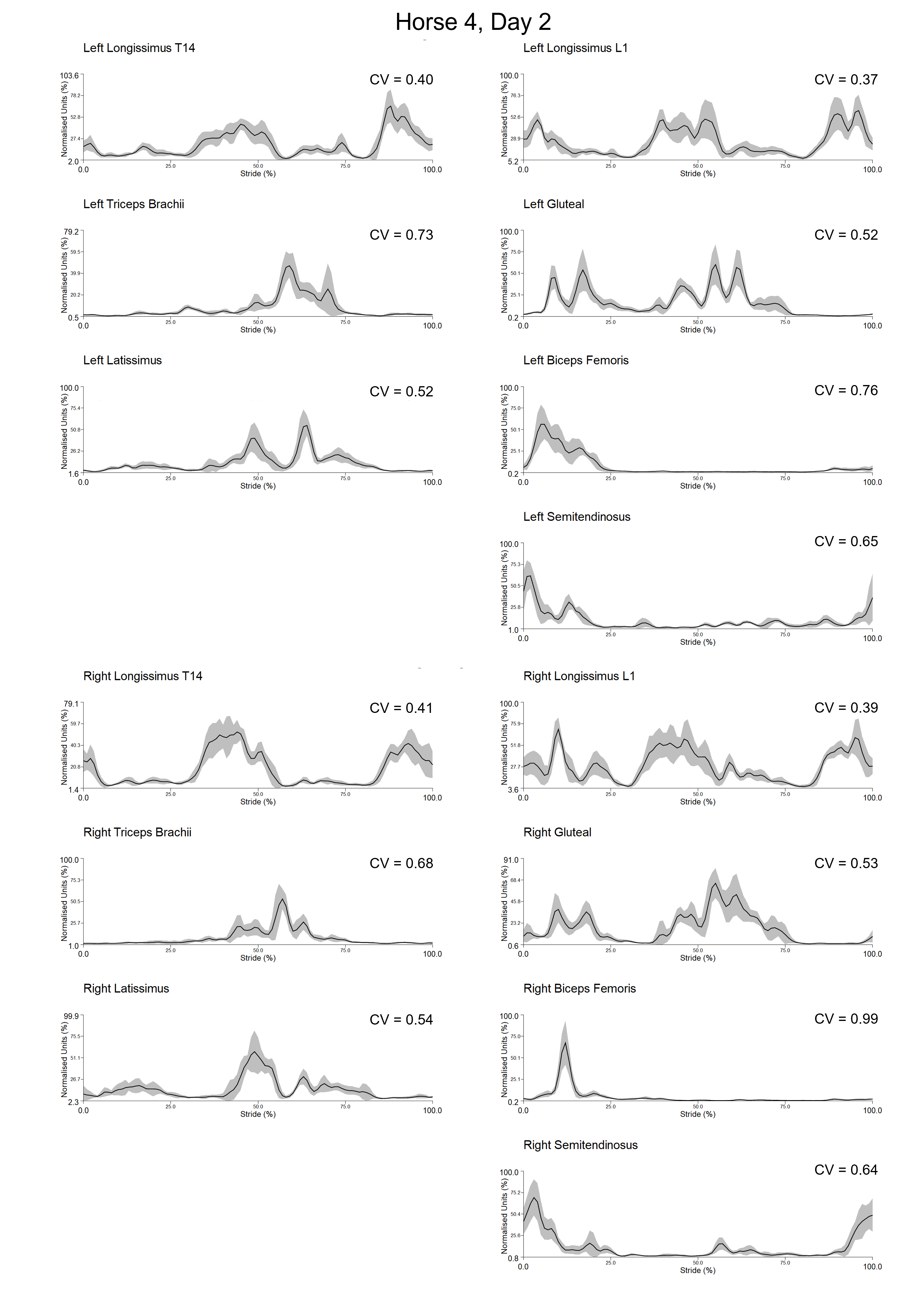

Supplement: S1 Fig — Mean (solid line) and standard deviation (grey shaded area) time and amplitude-normalised sEMG data from 9 and 8 trot strides are presented for left and right muscles, respectively. Coefficient of variation (CV) is indicated for each muscle. (TIF) [file pone.0288664.s003.tif]

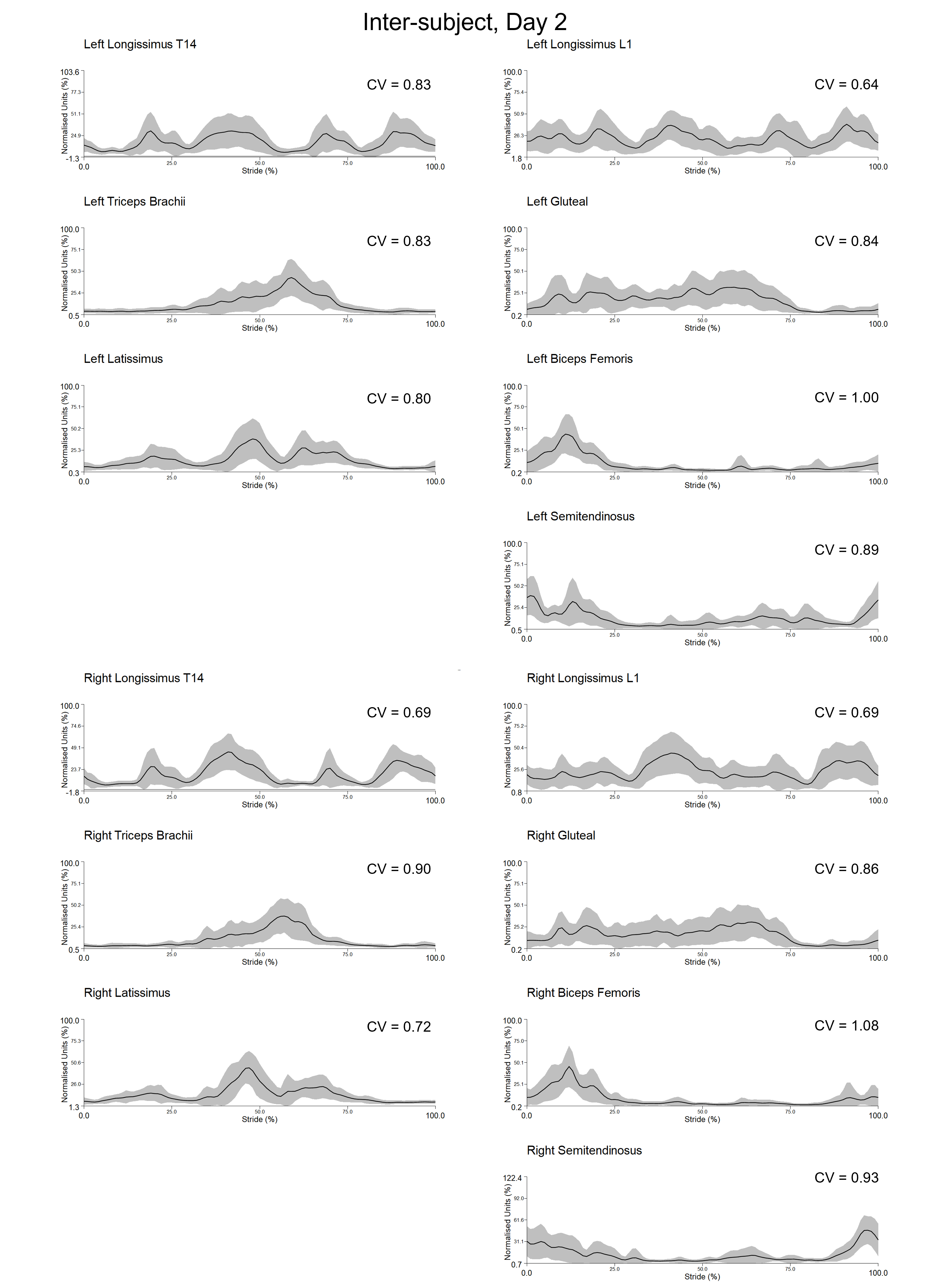

Supplement: S2 Fig — Mean (solid line) and standard deviation (grey shaded area) time and amplitude-normalised sEMG data from 74 and 67 trot strides are presented for left and right muscles, respectively. Coefficient of variation (CV) is indicated for each muscle. (TIF) [file pone.0288664.s004.tif]

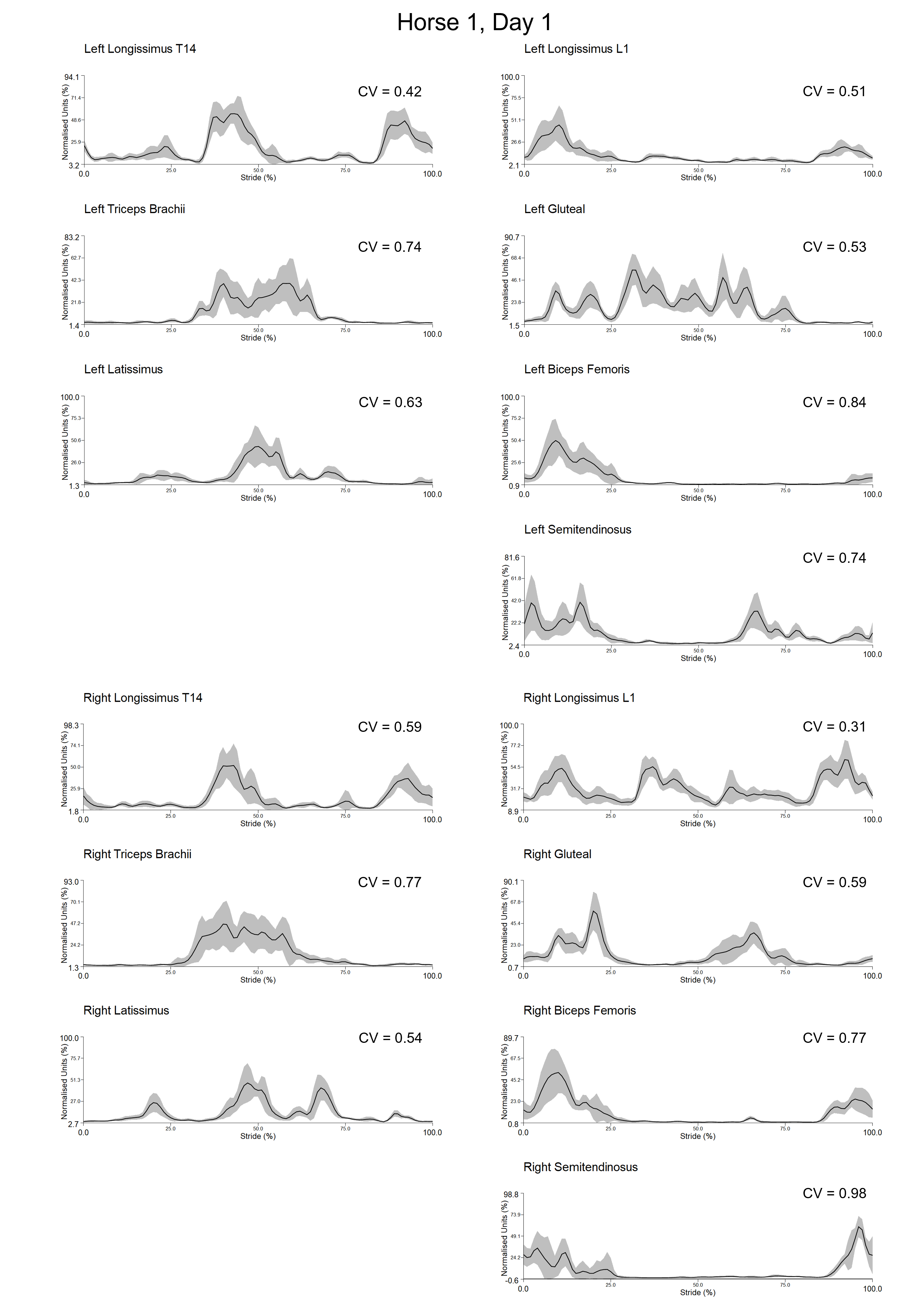

Supplement: S3 Fig — Mean (solid line) and standard deviation (grey shaded area) time and amplitude-normalised sEMG data from 10 trot strides are presented for each muscle. Coefficient of variation (CV) is indicated for each muscle. (TIF) [file pone.0288664.s005.tif]

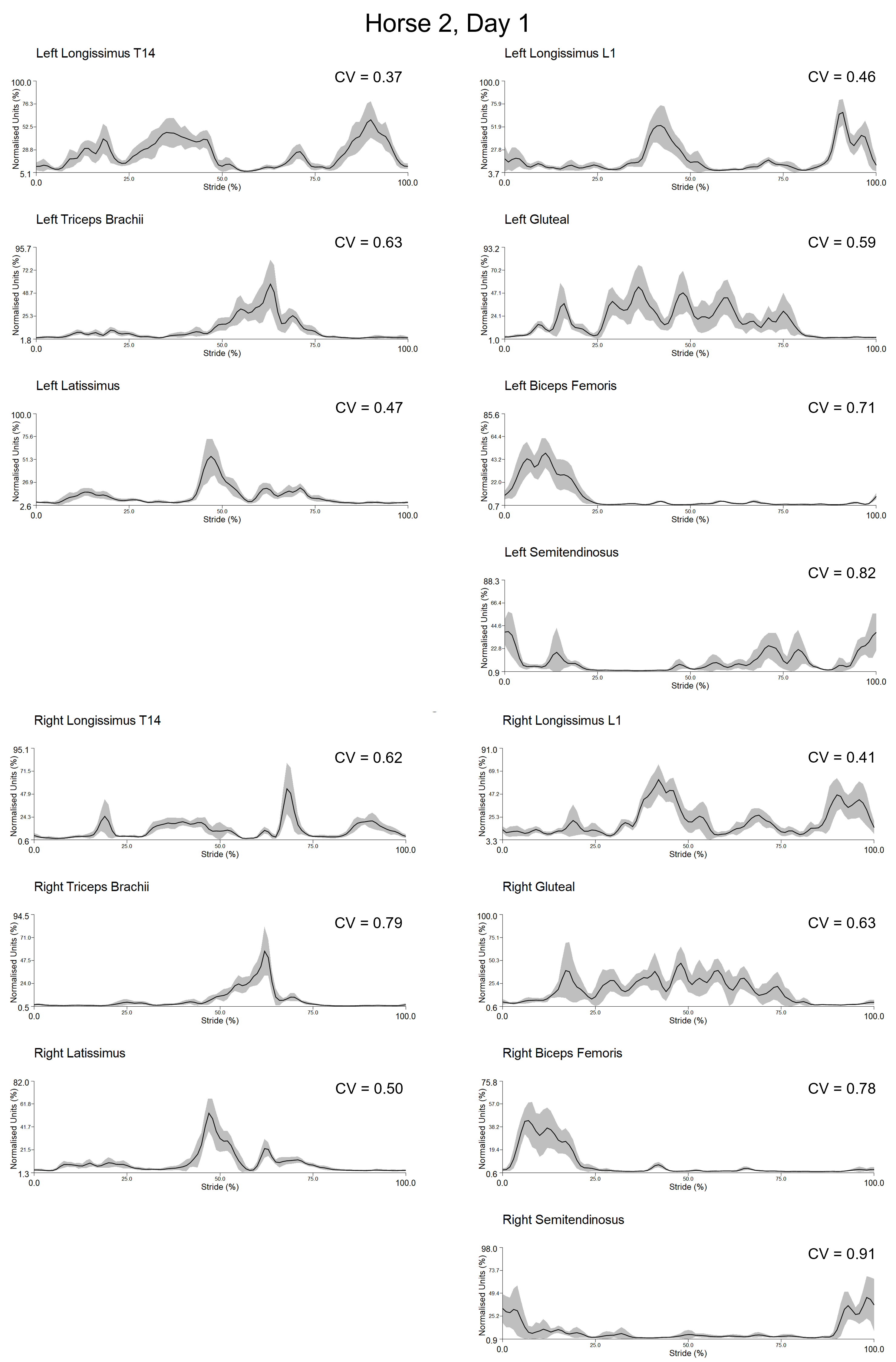

Supplement: S4 Fig — Mean (solid line) and standard deviation (grey shaded area) time and amplitude-normalised sEMG data from 10 trot strides are presented for each muscle. Coefficient of variation (CV) is indicated for each muscle. (TIF) [file pone.0288664.s006.tif]

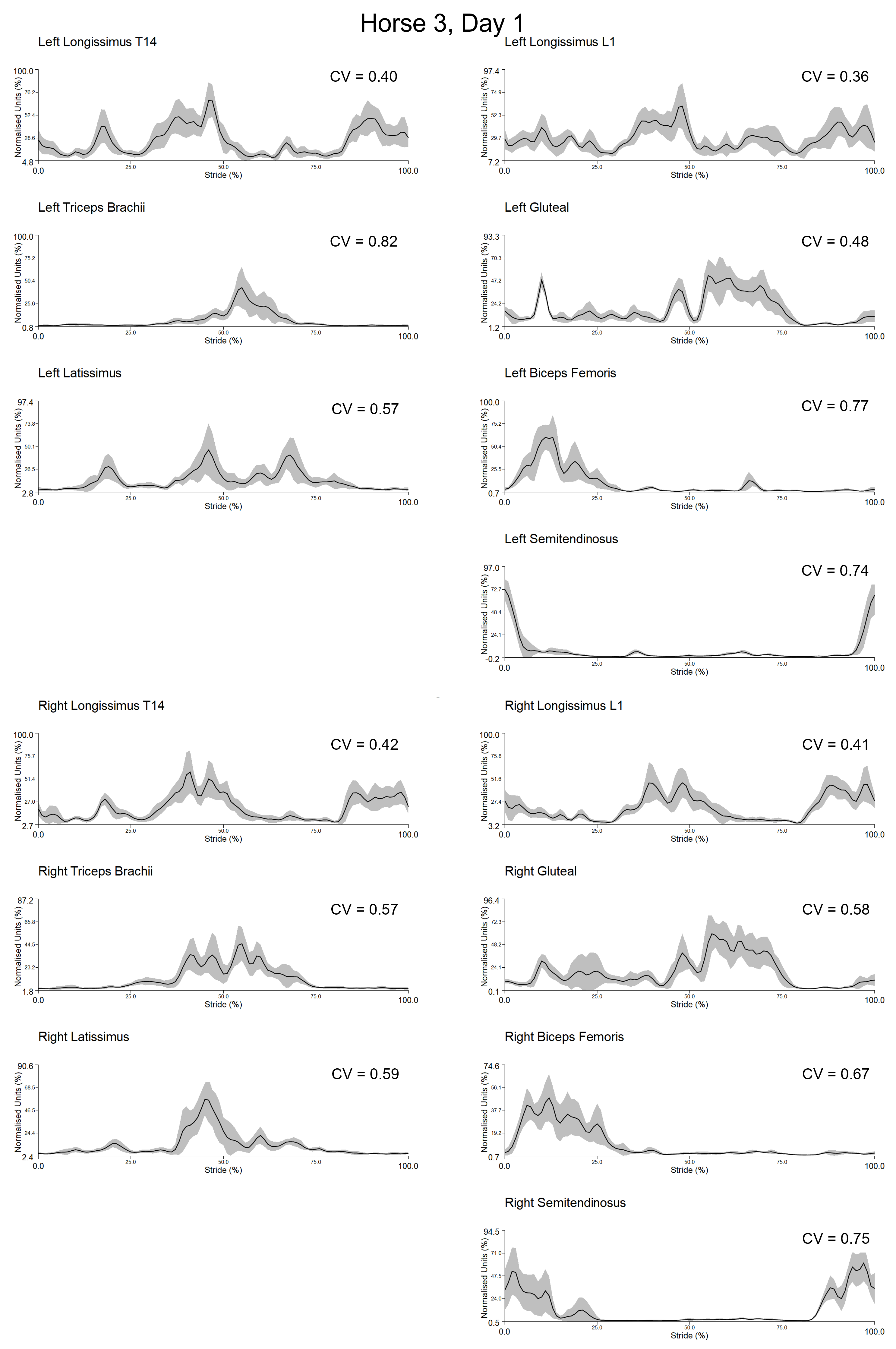

Supplement: S5 Fig — Mean (solid line) and standard deviation (grey shaded area) time and amplitude-normalised sEMG data from 10 trot strides are presented for each muscle. Coefficient of variation (CV) is indicated for each muscle. (TIF) [file pone.0288664.s007.tif]

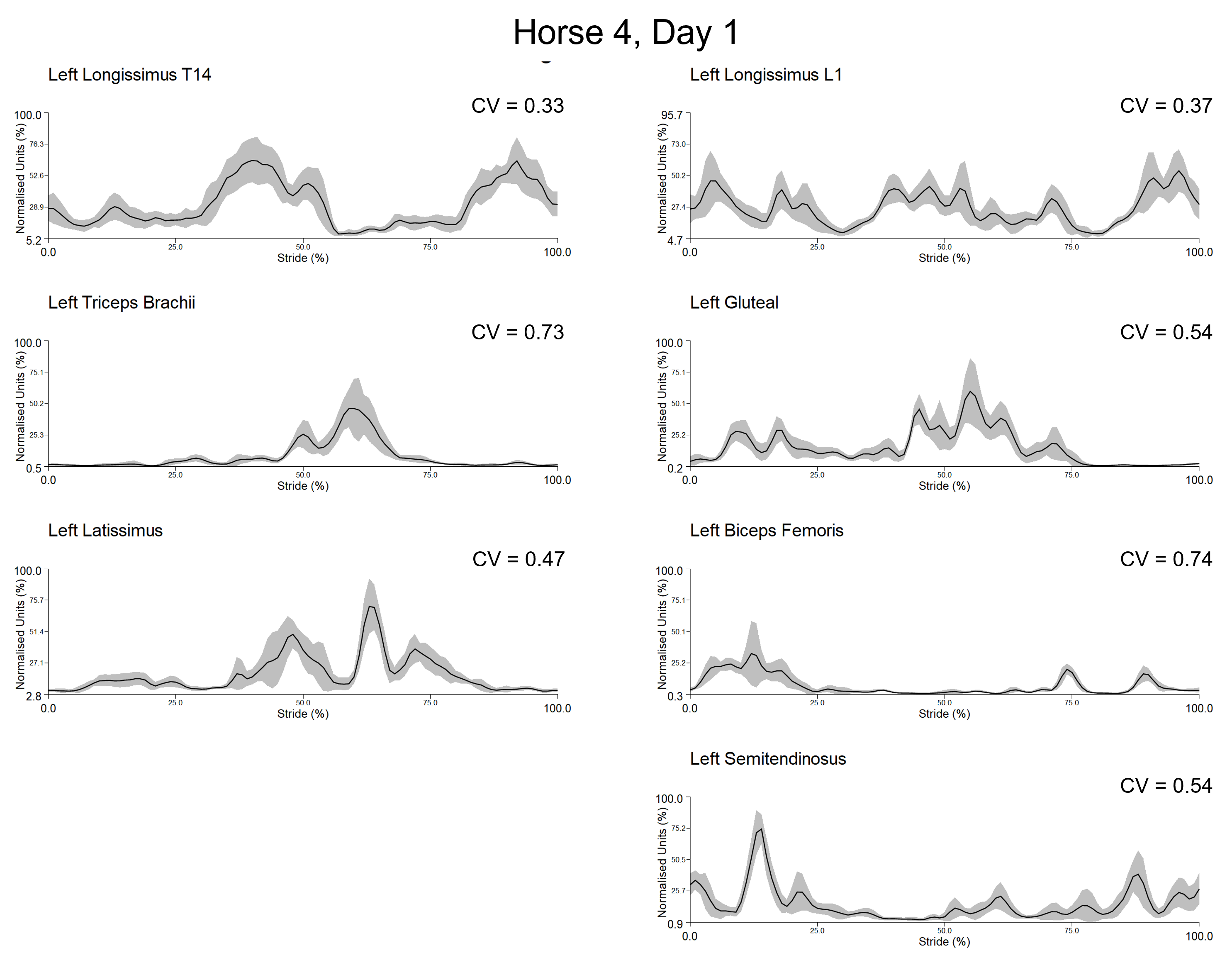

Supplement: S6 Fig — Mean (solid line) and standard deviation (grey shaded area) time and amplitude-normalised sEMG data from 10 trot strides are presented for each muscle. Coefficient of variation (CV) is indicated for each muscle. (TIF) [file pone.0288664.s008.tif]

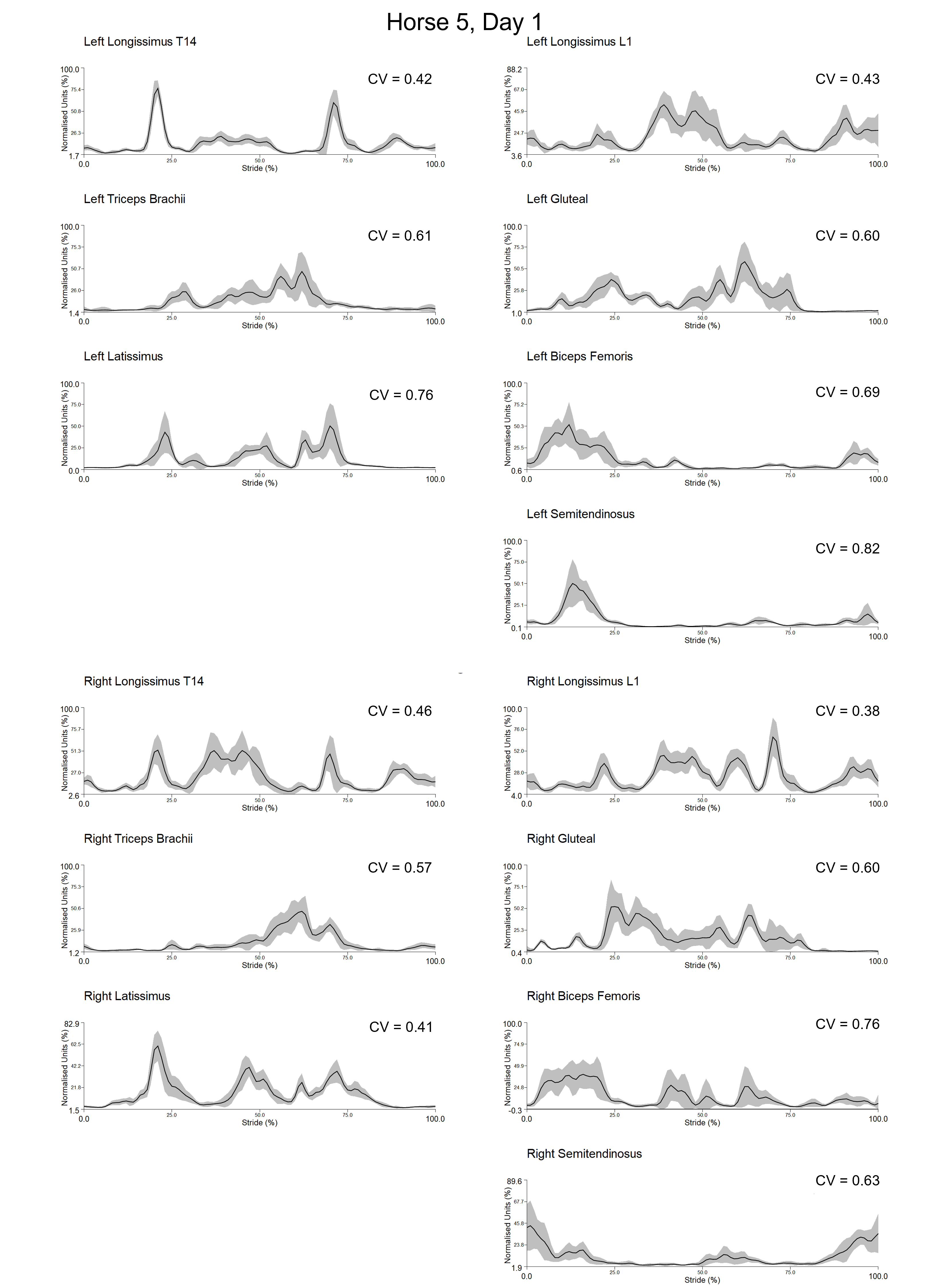

Supplement: S7 Fig — Mean (solid line) and standard deviation (grey shaded area) time and amplitude-normalised sEMG data from 9 and 10 trot strides are presented for left and right muscles, respectively. Coefficient of variation (CV) is indicated for each muscle. (TIF) [file pone.0288664.s009.tif]

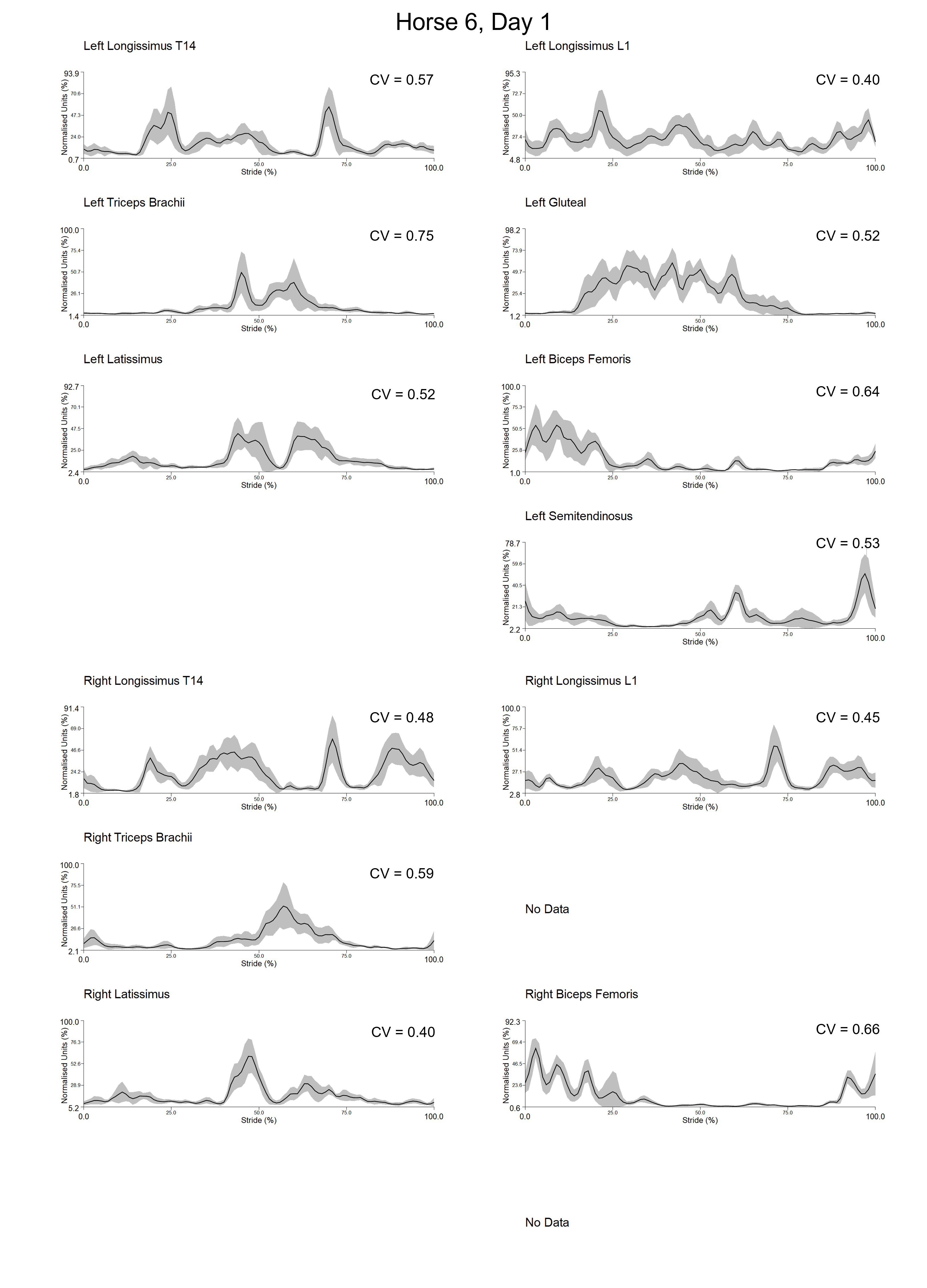

Supplement: S8 Fig — Mean (solid line) and standard deviation (grey shaded area) time and amplitude-normalised sEMG data from 9 and 10 trot strides are presented for left and right muscles, respectively. Coefficient of variation (CV) is indicated for each muscle. (TIF) [file pone.0288664.s010.tif]

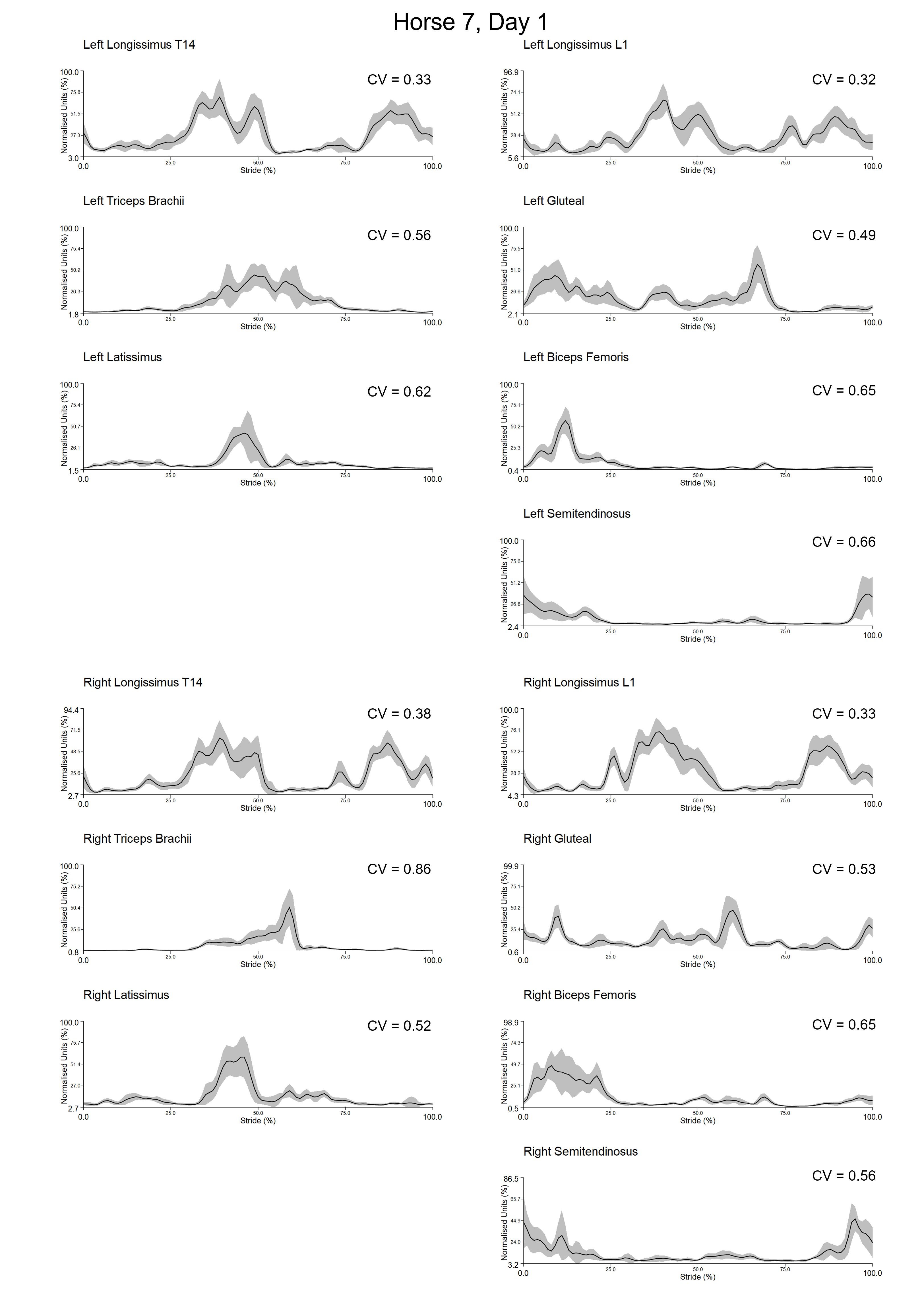

Supplement: S9 Fig — Mean (solid line) and standard deviation (grey shaded area) time and amplitude-normalised sEMG data from 10 trot strides are presented each muscle. Coefficient of variation (CV) is indicated for each muscle. (TIF) [file pone.0288664.s011.tif]

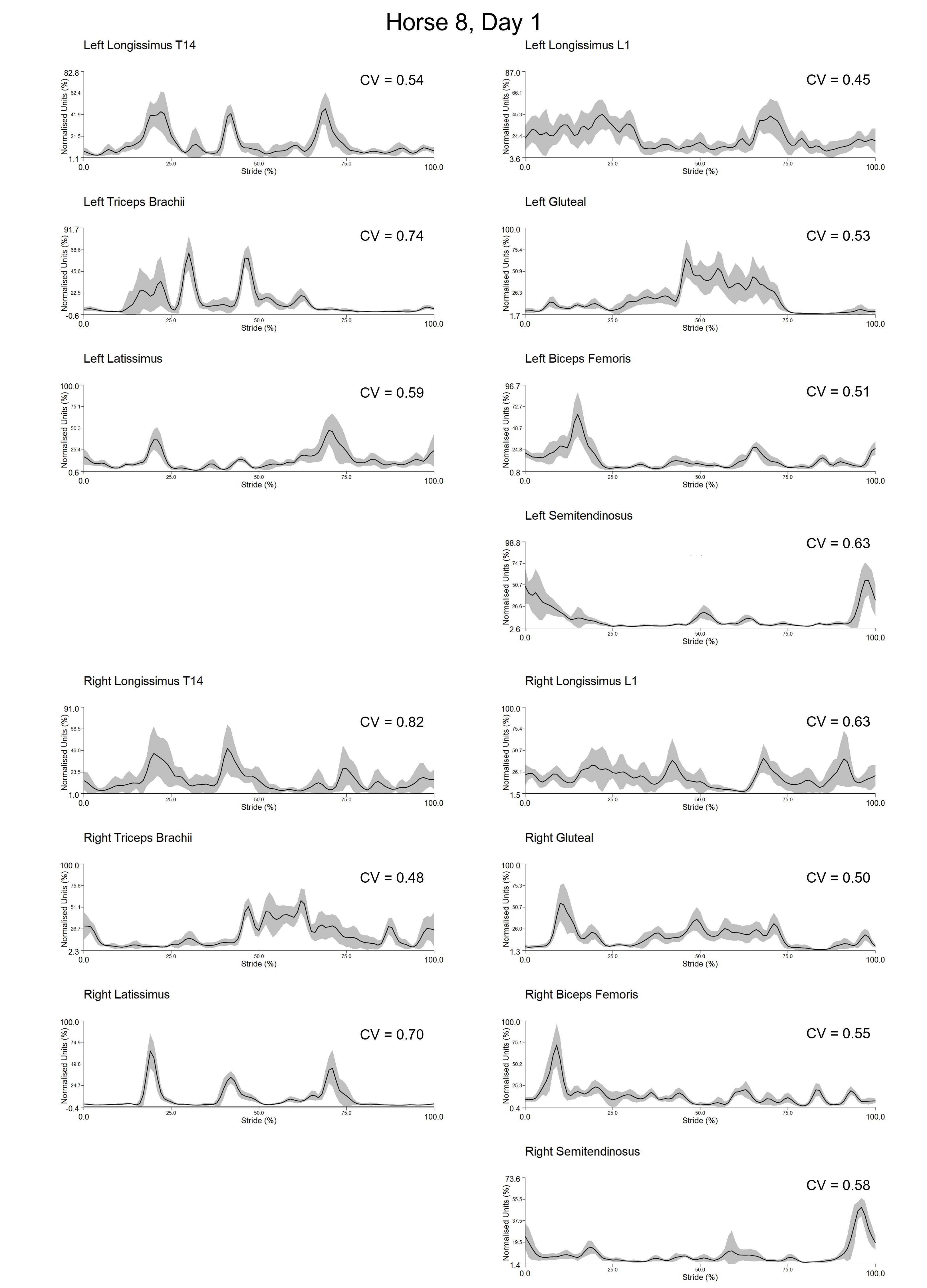

Supplement: S10 Fig — Mean (solid line) and standard deviation (grey shaded area) time and amplitude-normalised sEMG data from 10 trot strides are presented each muscle. Coefficient of variation (CV) is indicated for each muscle. (TIF) [file pone.0288664.s012.tif]

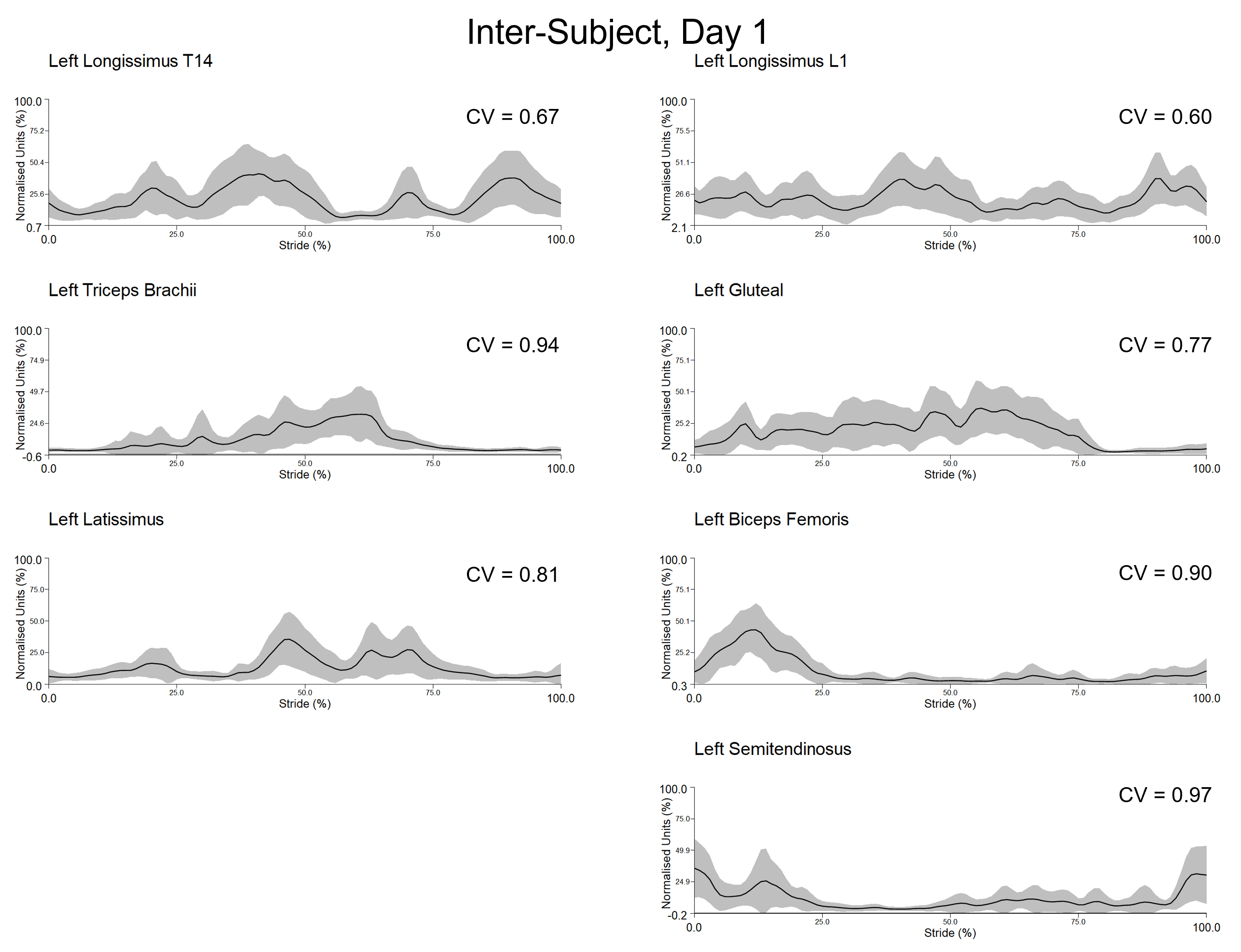

Supplement: S11 Fig — Mean (solid line) and standard deviation (grey shaded area) time and amplitude-normalised sEMG data from 78 trot strides are presented. Coefficient of variation (CV) is indicated for each muscle. (TIF) [file pone.0288664.s013.tif]

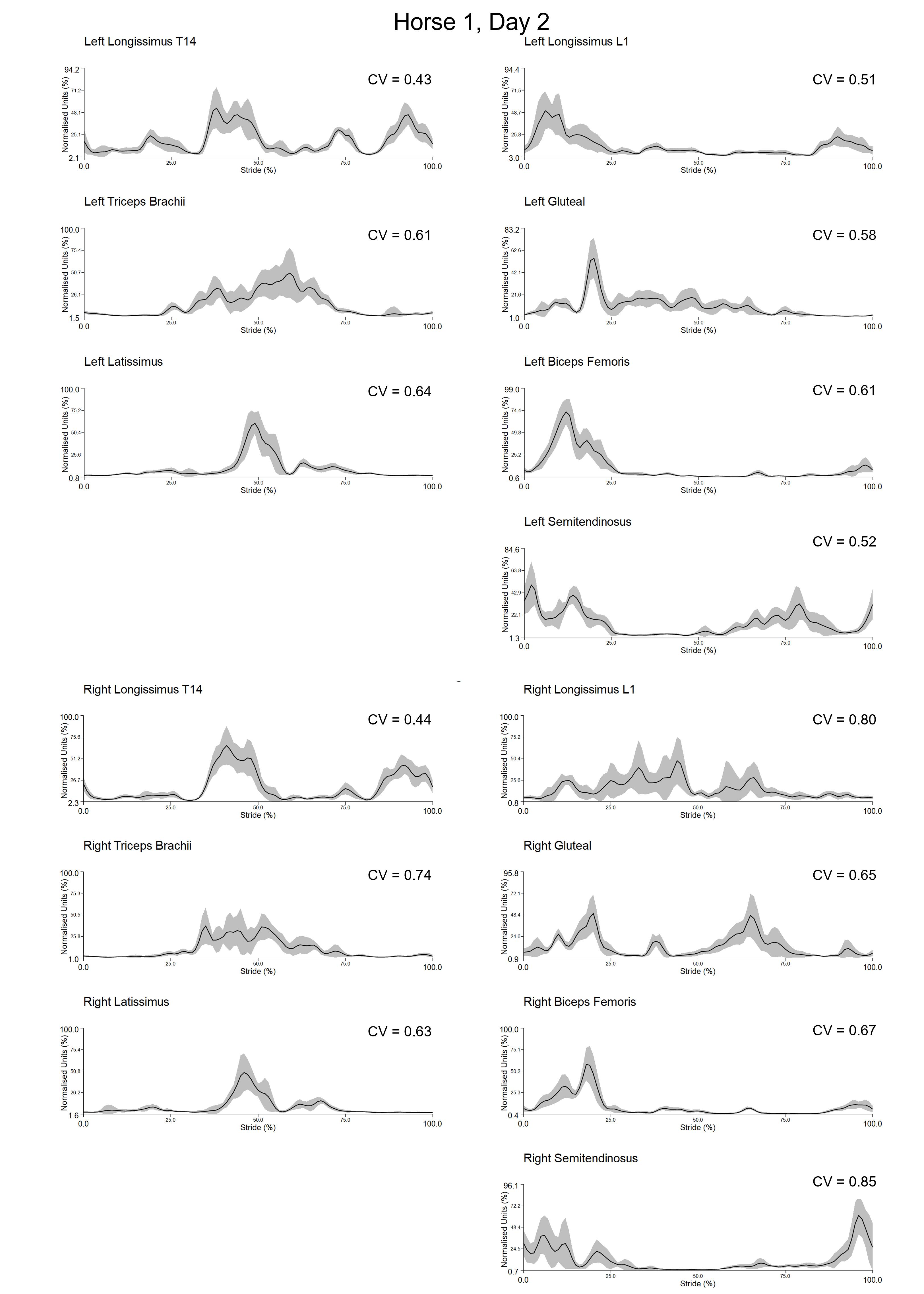

Supplement: S12 Fig — Mean (solid line) and standard deviation (grey shaded area) time and amplitude-normalised sEMG data from 10 trot strides are presented each muscle. Coefficient of variation (CV) is indicated for each muscle. (TIF) [file pone.0288664.s014.tif]

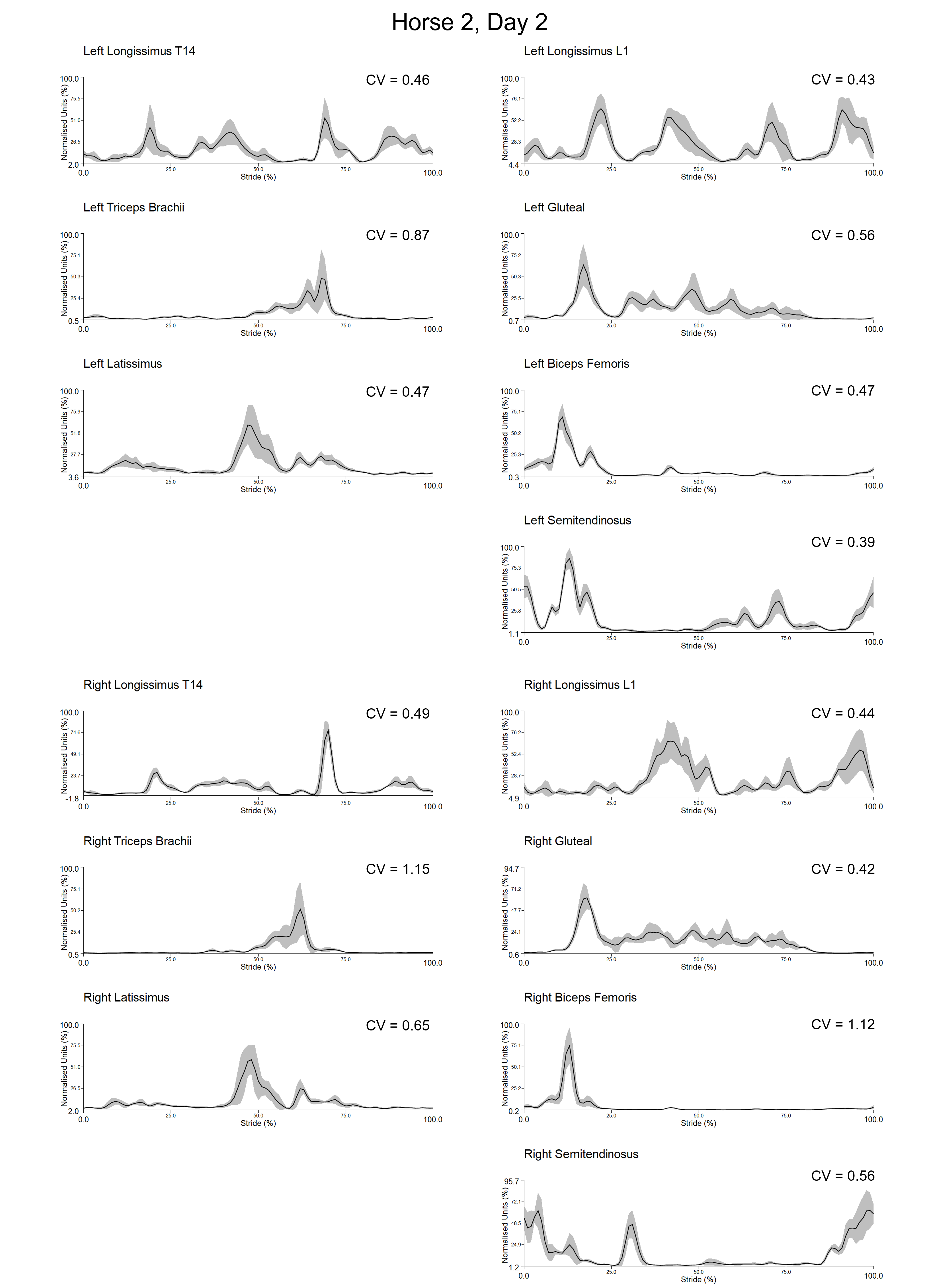

Supplement: S13 Fig — Mean (solid line) and standard deviation (grey shaded area) time and amplitude-normalised sEMG data from 8 and 6 trot strides are presented for left and right muscles, respectively. Coefficient of variation (CV) is indicated for each muscle. (TIF) [file pone.0288664.s015.tif]

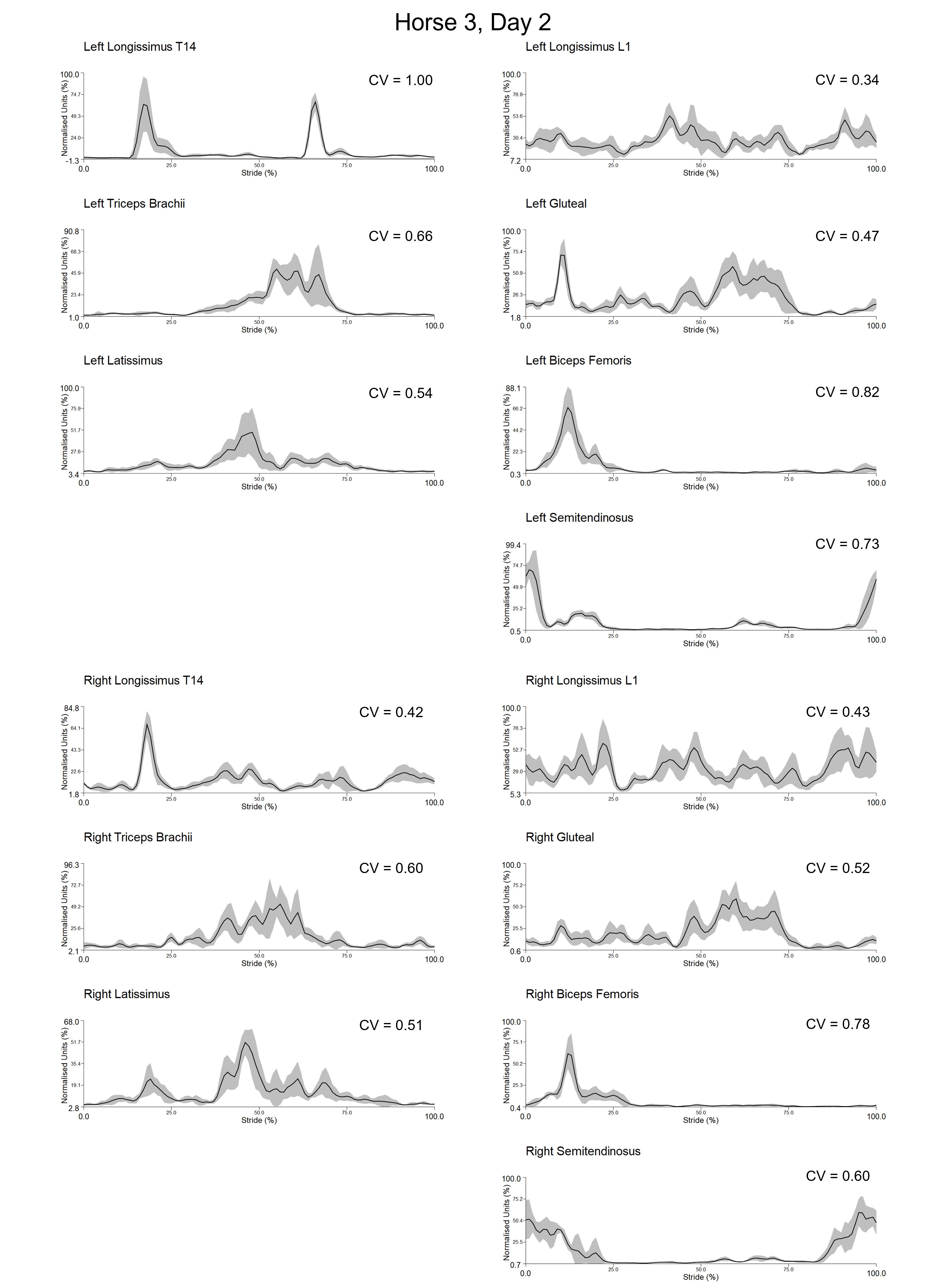

Supplement: S14 Fig — Mean (solid line) and standard deviation (grey shaded area) time and amplitude-normalised sEMG data from 7 and 6 trot strides are presented for left and right muscles, respectively. Coefficient of variation (CV) is indicated for each muscle. (TIF) [file pone.0288664.s016.tif]

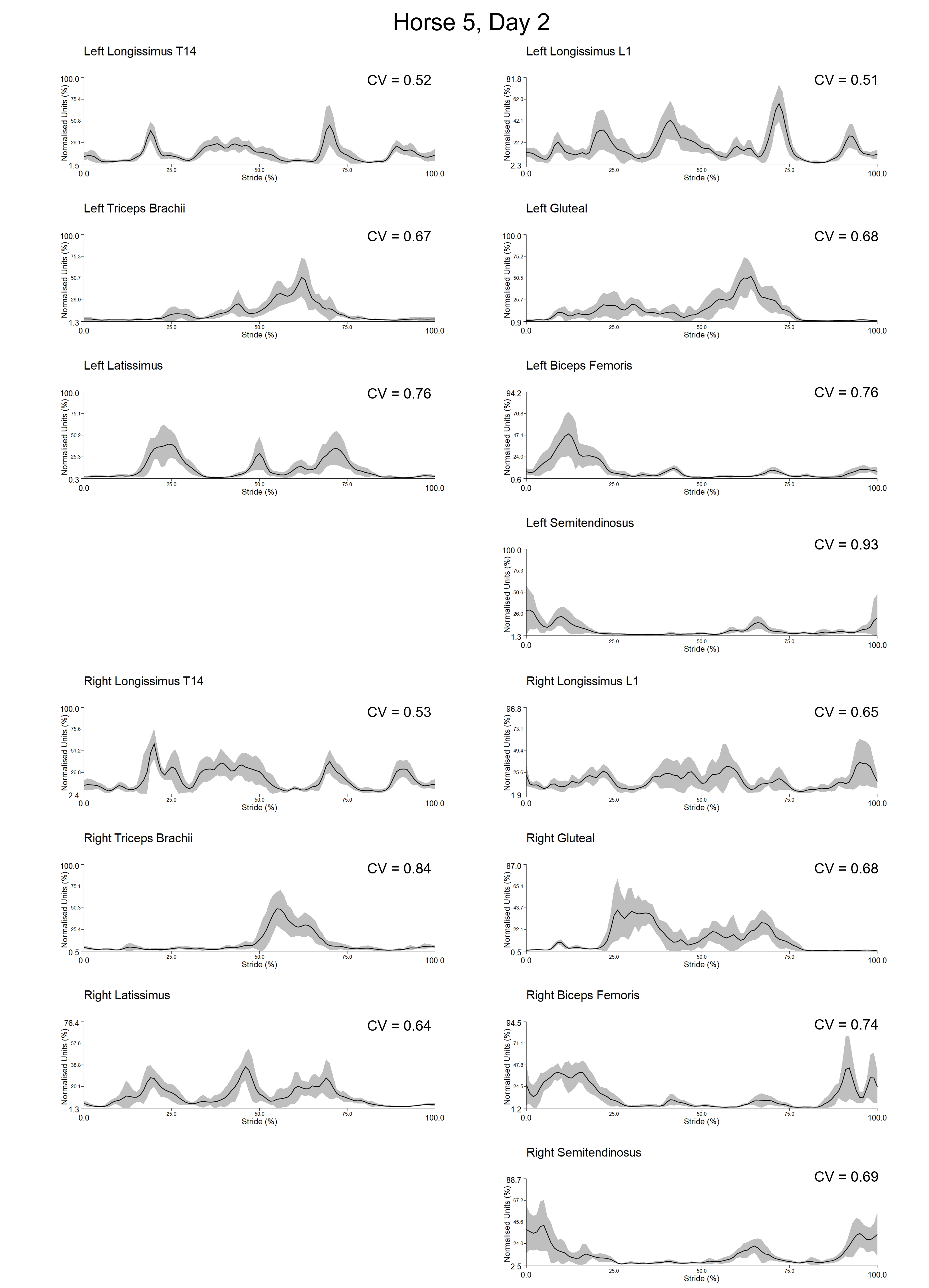

Supplement: S15 Fig — Mean (solid line) and standard deviation (grey shaded area) time and amplitude-normalised sEMG data from 10 and 8 trot strides are presented for left and right muscles, respectively. Coefficient of variation (CV) is indicated for each muscle. (TIF) [file pone.0288664.s017.tif]

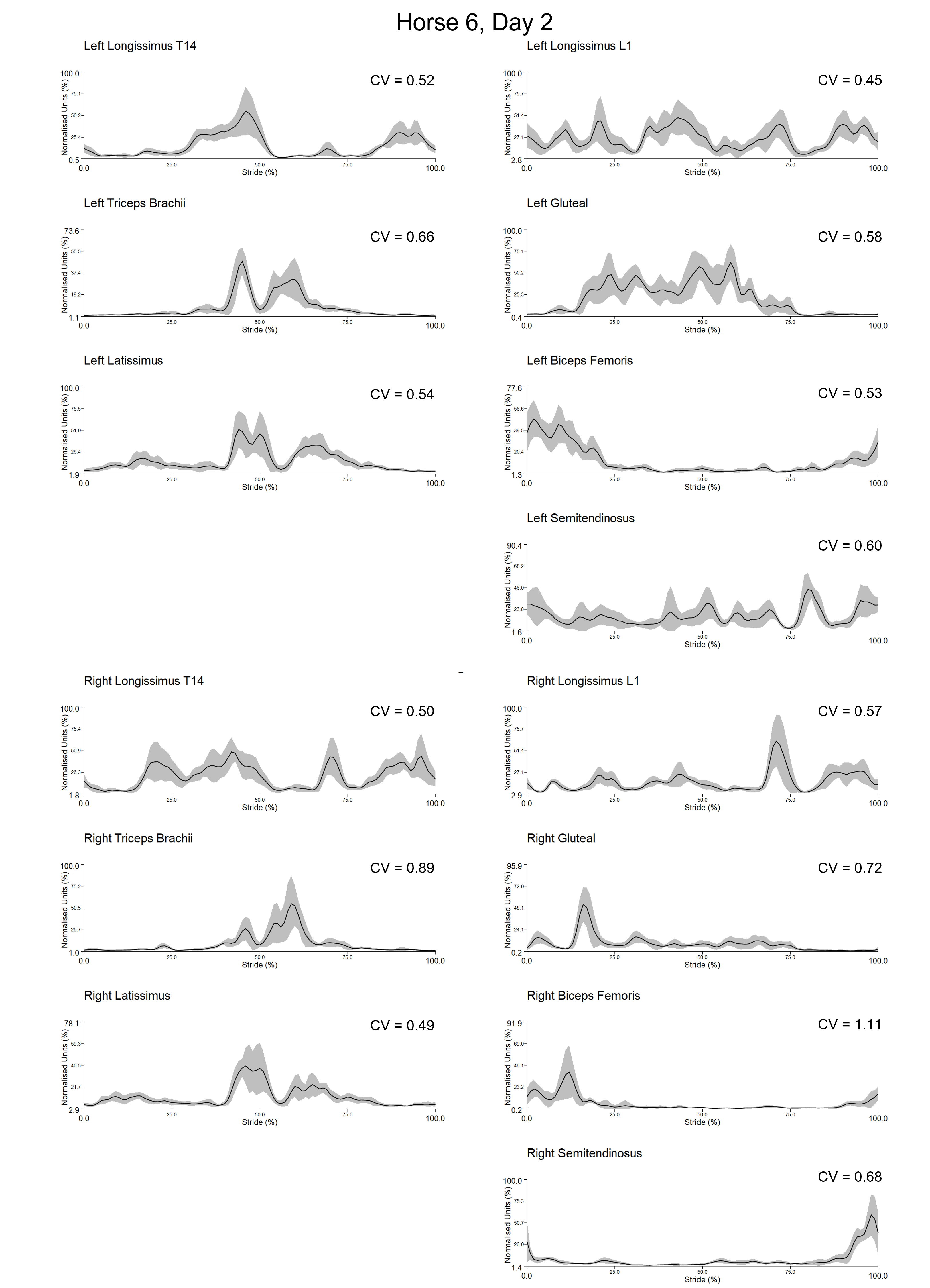

Supplement: S16 Fig — Mean (solid line) and standard deviation (grey shaded area) time and amplitude-normalised sEMG data from 10 and 9 trot strides are presented for left and right muscles, respectively. Coefficient of variation (CV) is indicated for each muscle. (TIF) [file pone.0288664.s018.tif]

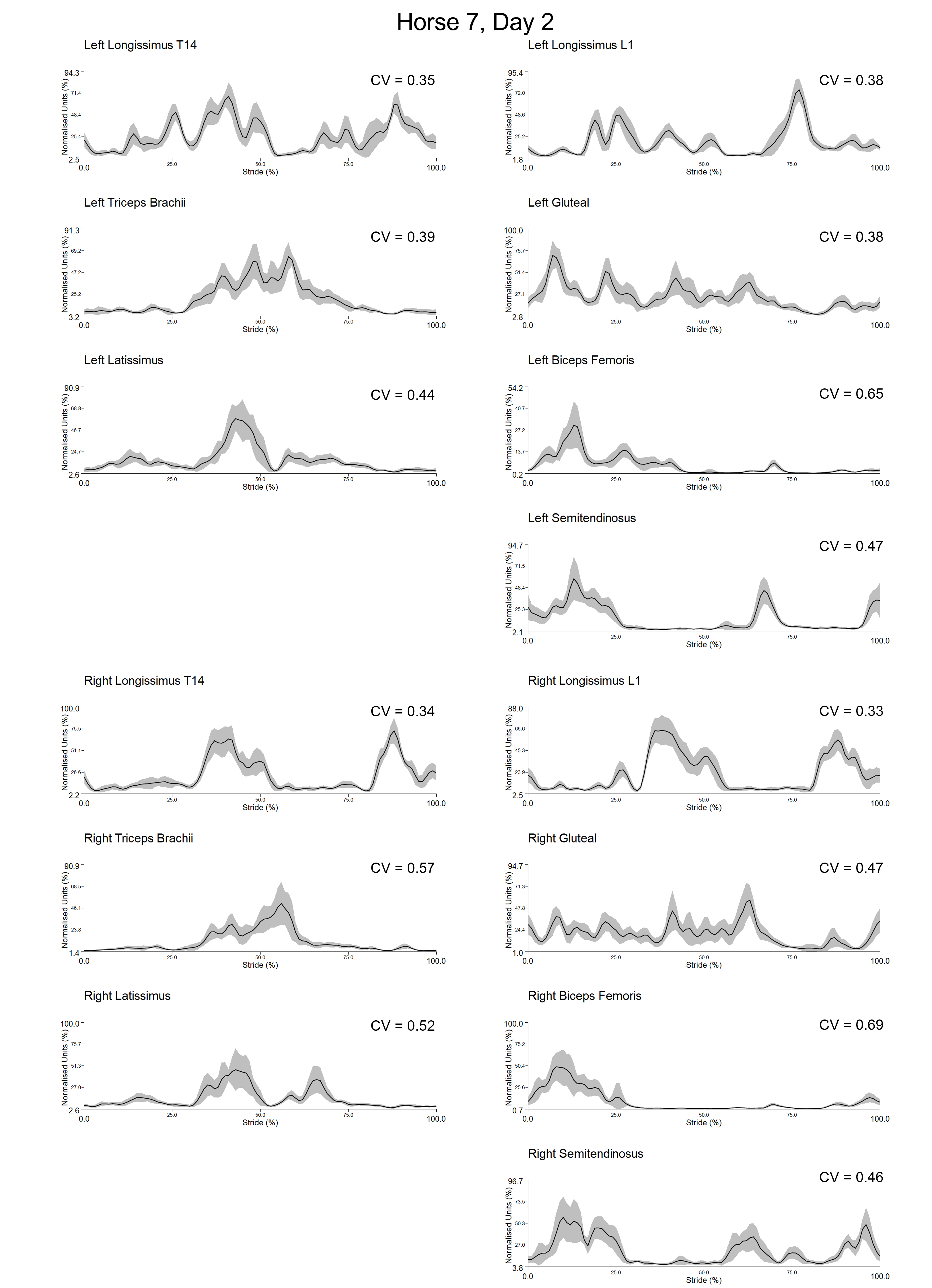

Supplement: S17 Fig — Mean (solid line) and standard deviation (grey shaded area) time and amplitude-normalised sEMG data from 10 trot strides are presented for each muscle. Coefficient of variation (CV) is indicated for each muscle. (TIF) [file pone.0288664.s019.tif]

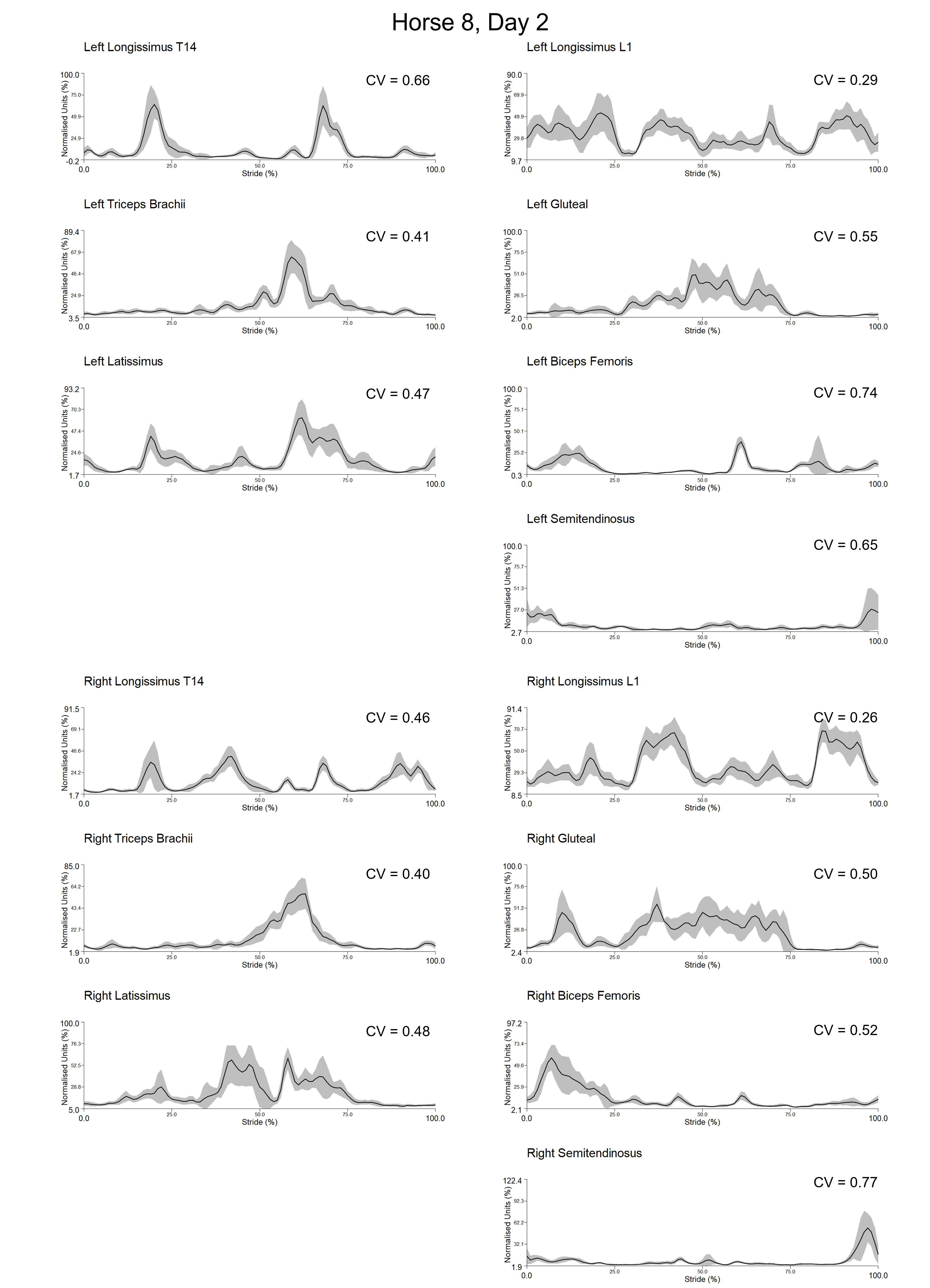

Supplement: S18 Fig — Mean (solid line) and standard deviation (grey shaded area) time and amplitude-normalised sEMG data from 10 trot strides are presented for each muscle. Coefficient of variation (CV) is indicated for each muscle. (TIF) [file pone.0288664.s020.tif]

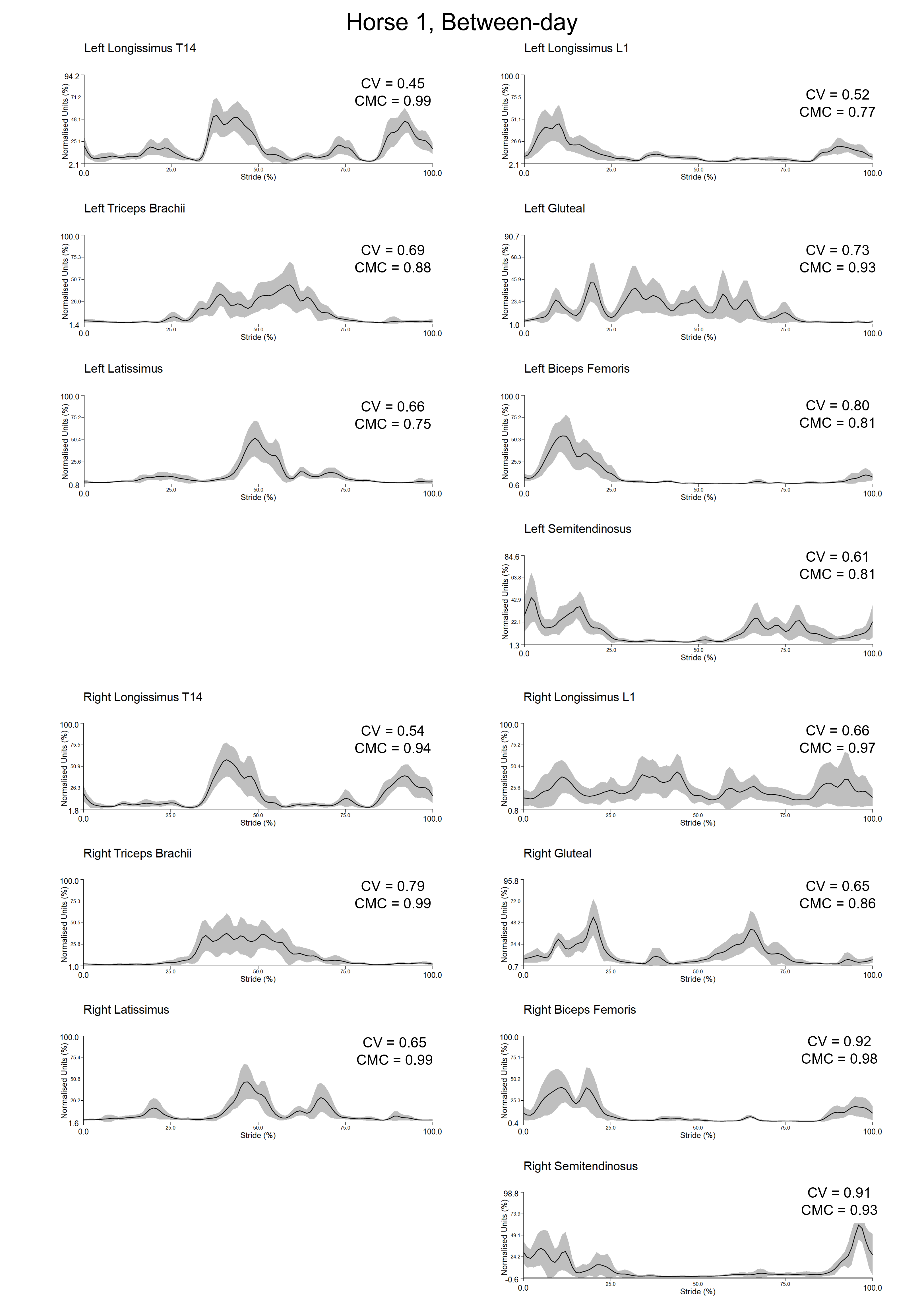

Supplement: S19 Fig — Mean (solid line) and standard deviation (grey shaded area) time and amplitude-normalised sEMG data from 20 trot strides are presented for each muscle. Coefficient of variation (CV) and coefficient of multiple correlation (CMC) is indicated for each muscle. (TIF) [file pone.0288664.s021.tif]

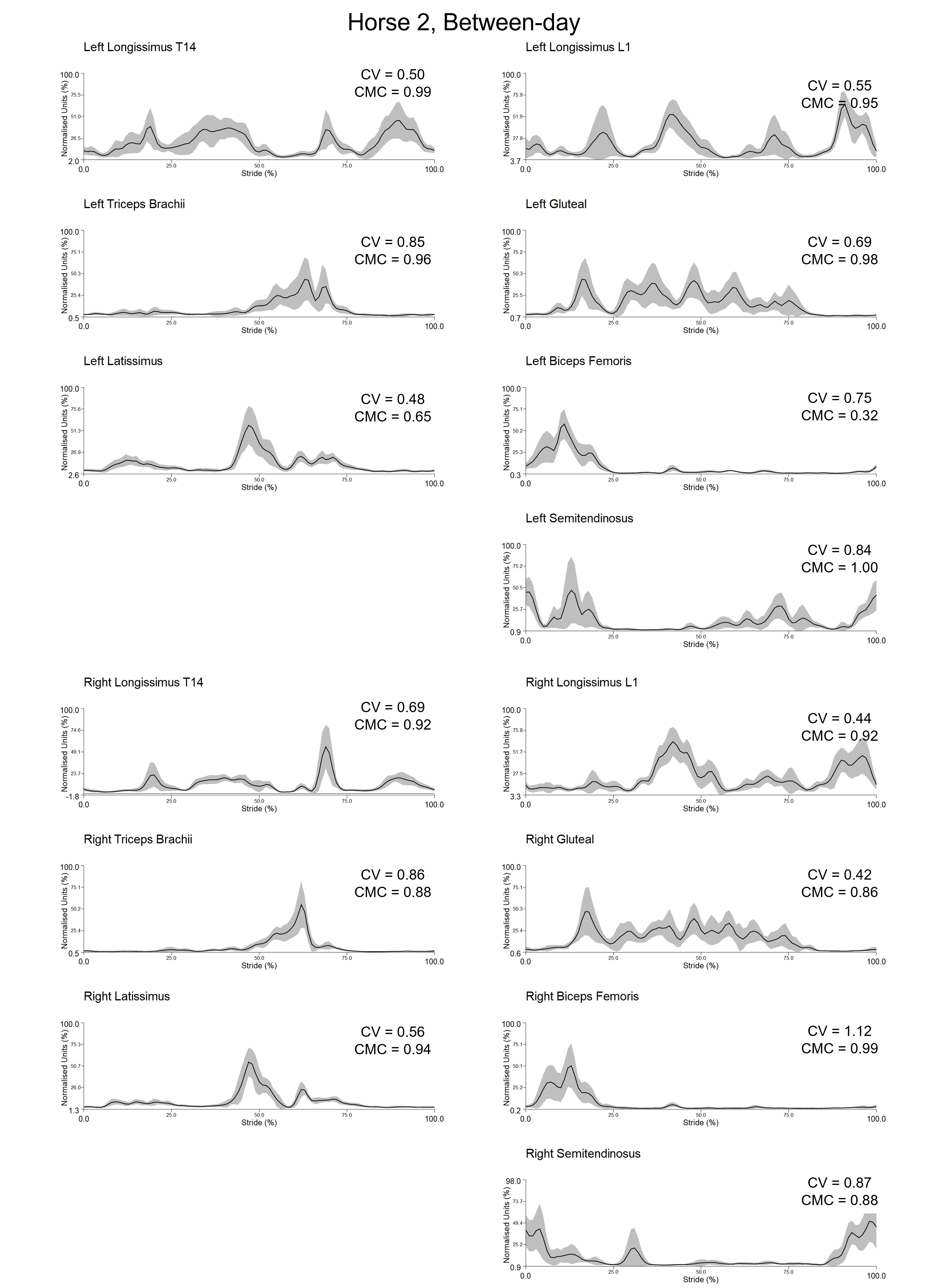

Supplement: S20 Fig — Mean (solid line) and standard deviation (grey shaded area) time and amplitude-normalised sEMG data from 18 and 16 trot strides are presented for left and right muscles, respectively. Coefficient of variation (CV) and coefficient of multiple correlation (CMC) is indicated for each muscle. (TIF) [file pone.0288664.s022.tif]

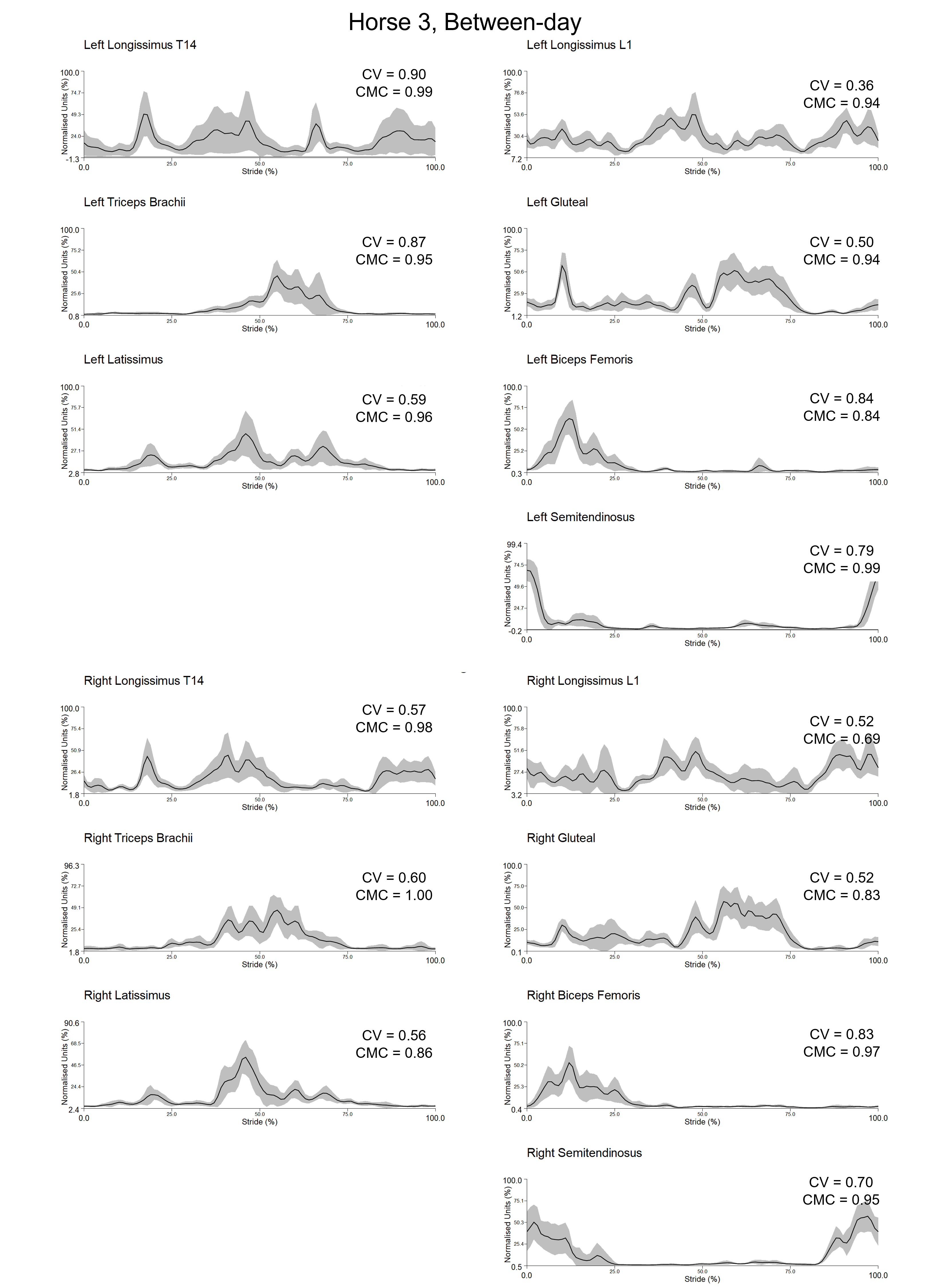

Supplement: S21 Fig — Mean (solid line) and standard deviation (grey shaded area) time and amplitude-normalised sEMG data from 17 and 16 trot strides are presented for left and right muscles, respectively. Coefficient of variation (CV) and coefficient of multiple correlation (CMC) is indicated for each muscle. (TIF) [file pone.0288664.s023.tif]

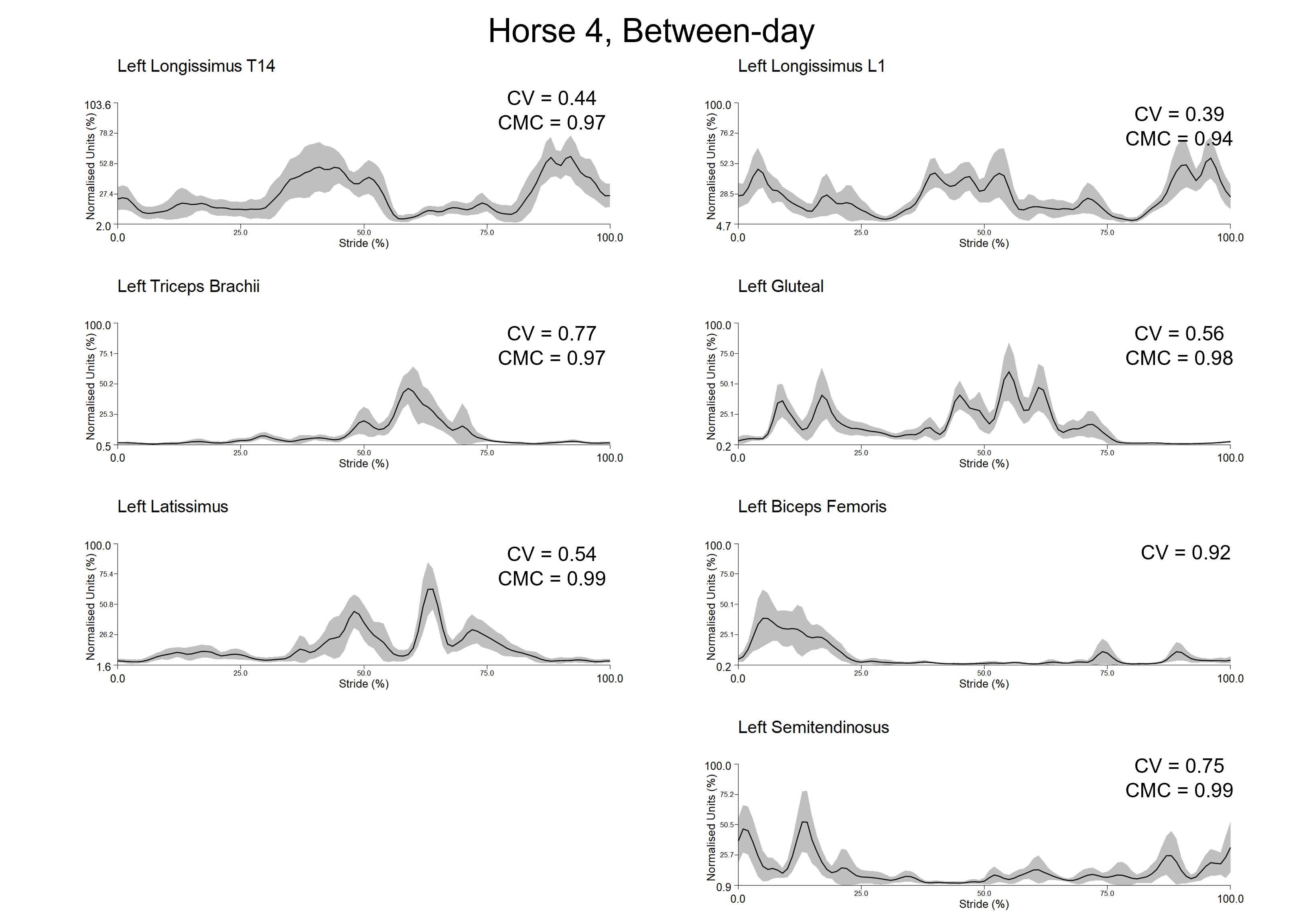

Supplement: S22 Fig — Mean (solid line) and standard deviation (grey shaded area) time and amplitude-normalised sEMG data from 19 trot strides are presented for each muscle. Coefficient of variation (CV) and coefficient of multiple correlation (CMC) is indicated for each muscle. (TIF) [file pone.0288664.s024.tif]

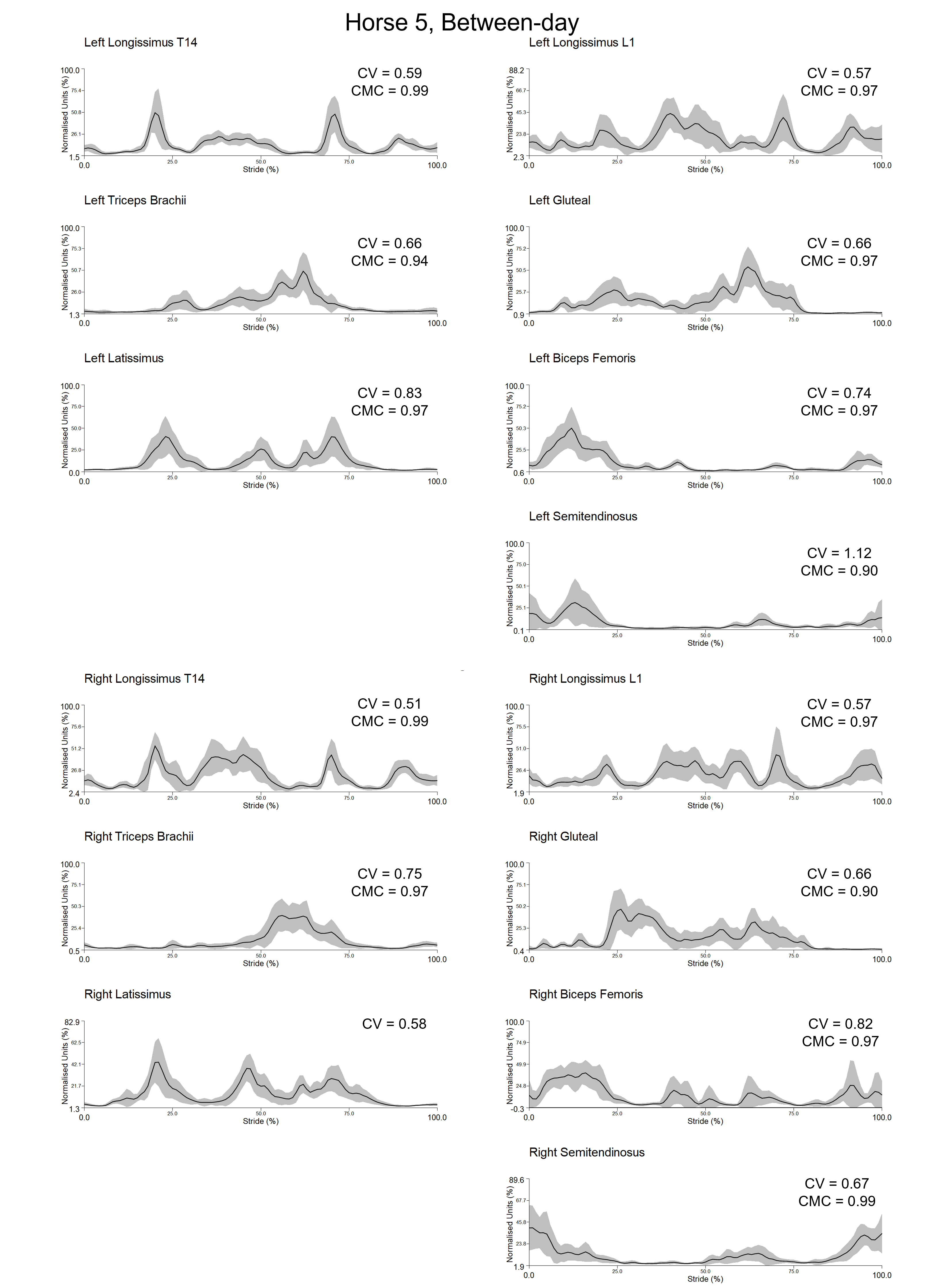

Supplement: S23 Fig — Mean (solid line) and standard deviation (grey shaded area) time and amplitude-normalised sEMG data from 19 and 18 trot strides are presented for left and right muscles, respectively. Coefficient of variation (CV) and coefficient of multiple correlation (CMC) is indicated for each muscle. (TIF) [file pone.0288664.s025.tif]

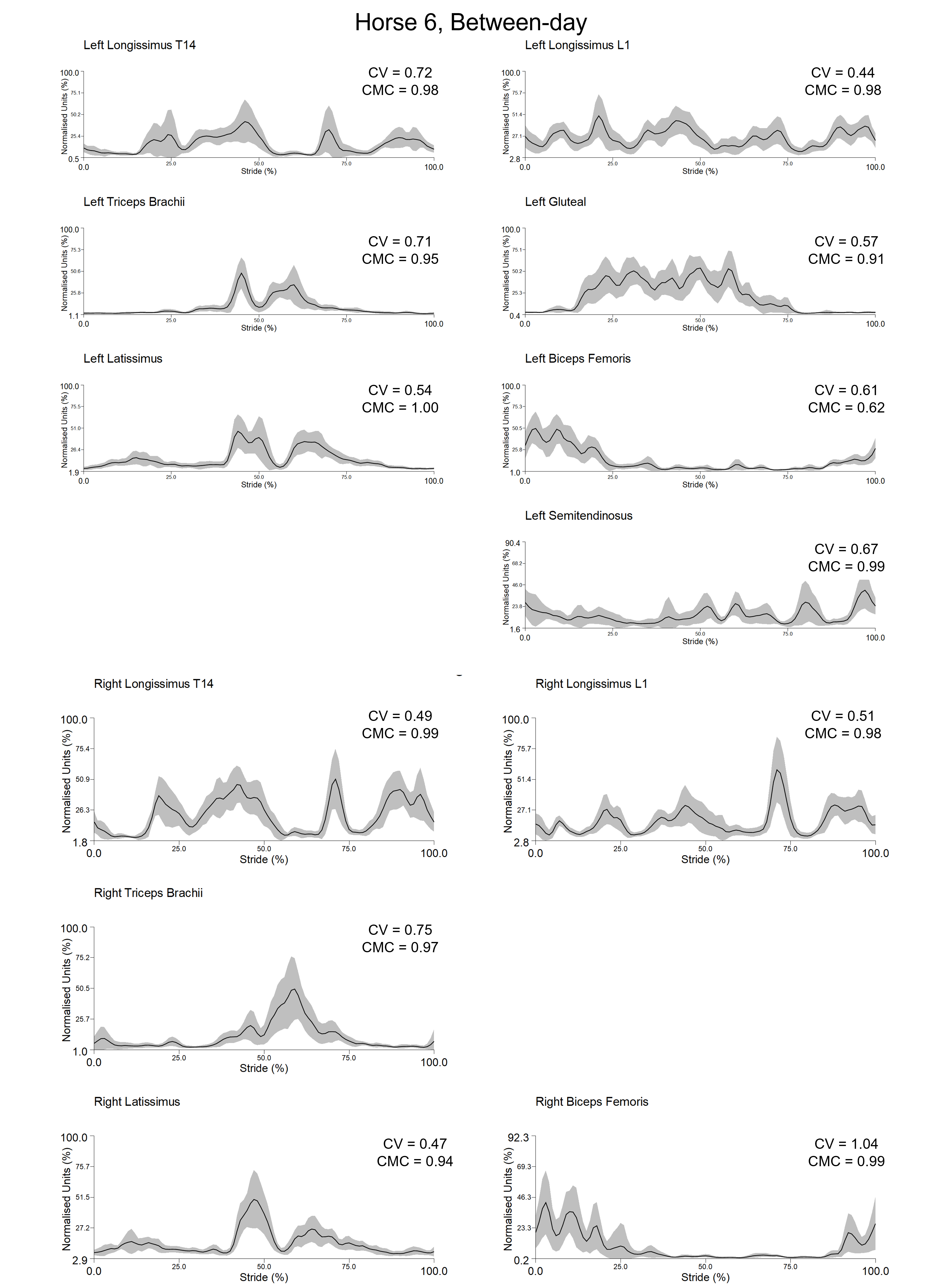

Supplement: S24 Fig — Mean (solid line) and standard deviation (grey shaded area) time and amplitude-normalised sEMG data from 19 trot strides are presented for each muscle. Coefficient of variation (CV) and coefficient of multiple correlation (CMC) is indicated for each muscle. (TIF) [file pone.0288664.s026.tif]

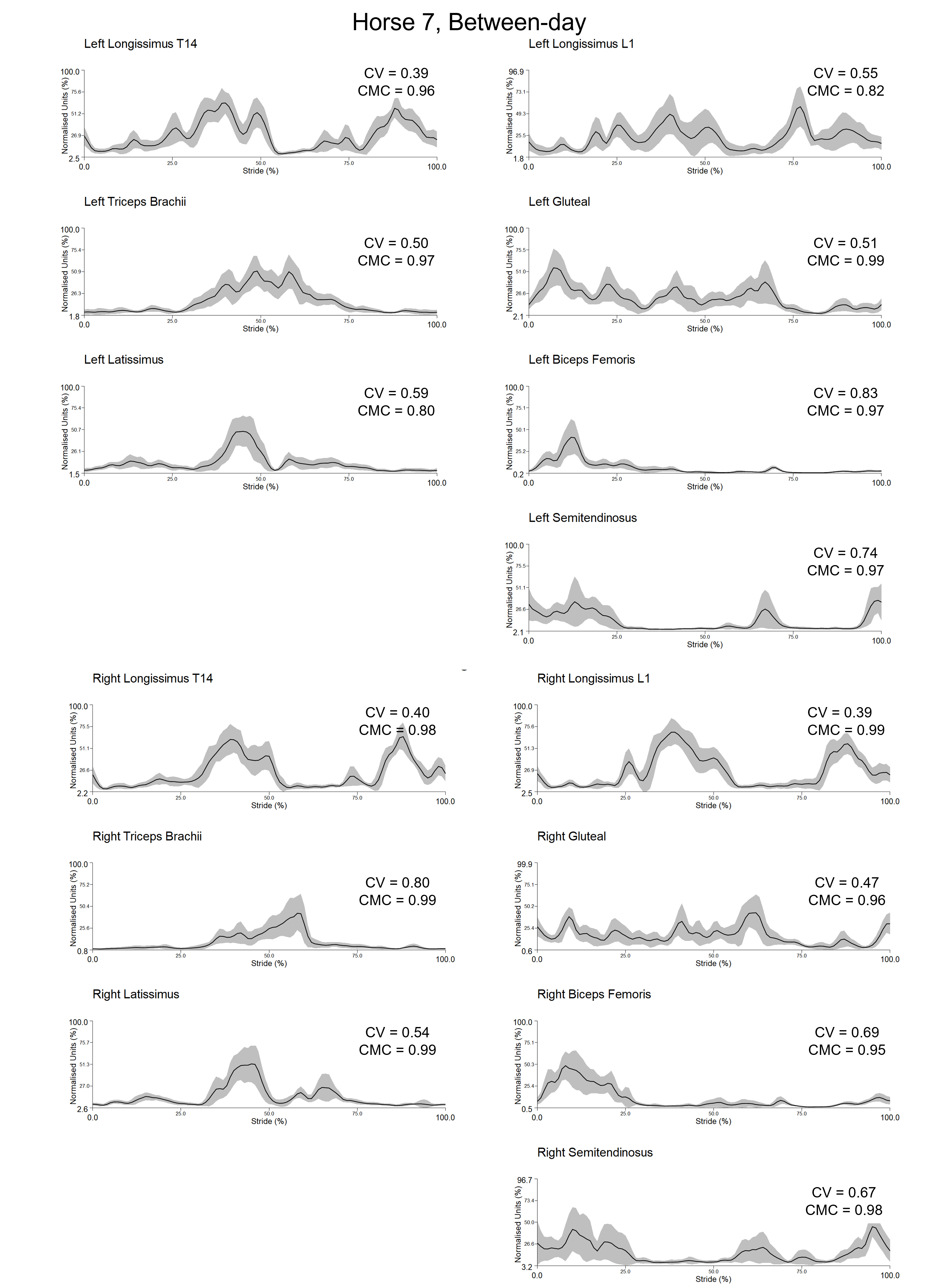

Supplement: S25 Fig — Mean (solid line) and standard deviation (grey shaded area) time and amplitude-normalised sEMG data from 20 trot strides are presented for each muscle. Coefficient of variation (CV) and coefficient of multiple correlation (CMC) is indicated for each muscle. (TIF) [file pone.0288664.s027.tif]

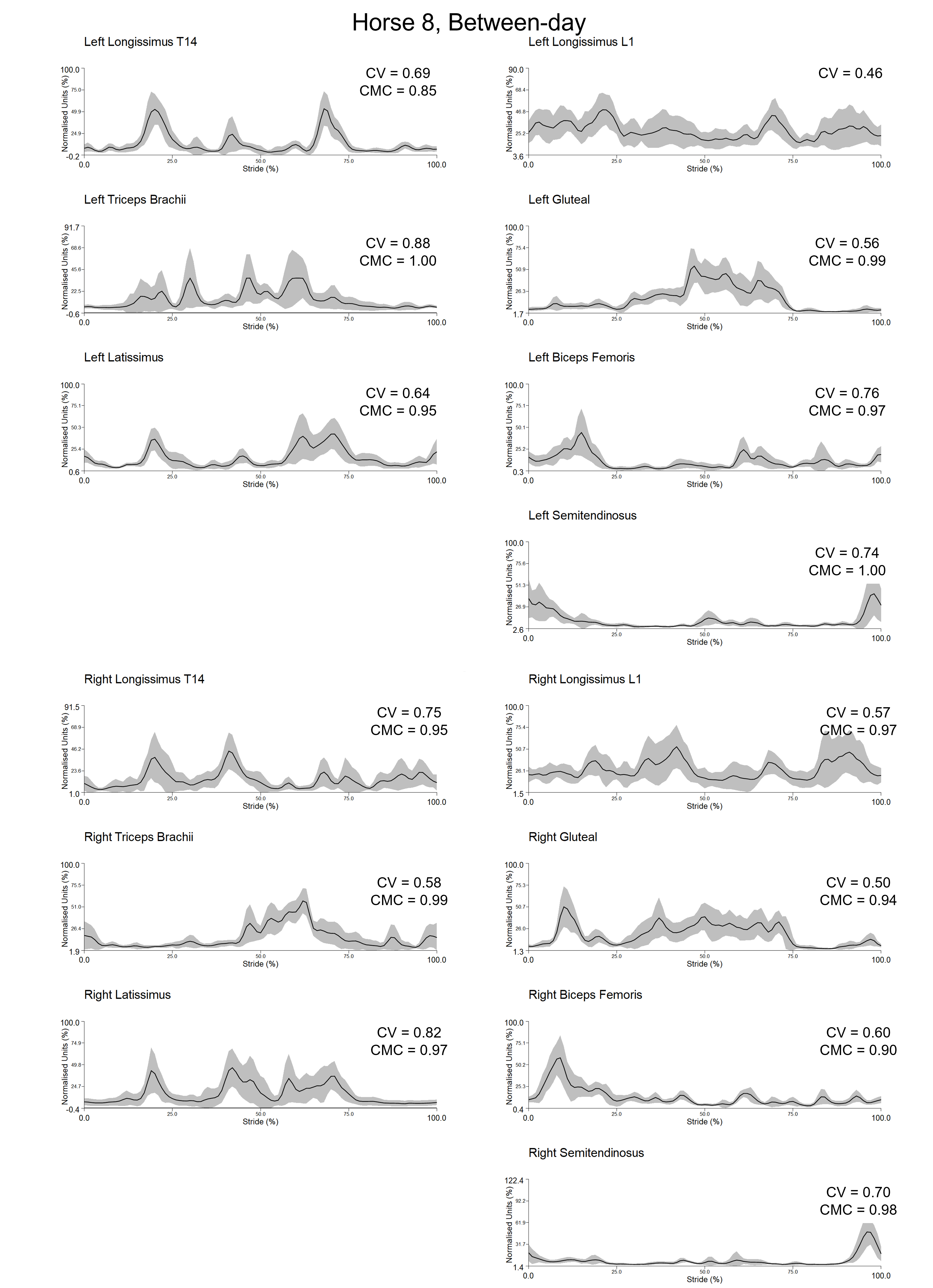

Supplement: S26 Fig — Mean (solid line) and standard deviation (grey shaded area) time and amplitude-normalised sEMG data from 20 trot strides are presented for each muscle. Coefficient of variation (CV) is indicated for each muscle. (TIF) [file pone.0288664.s028.tif]

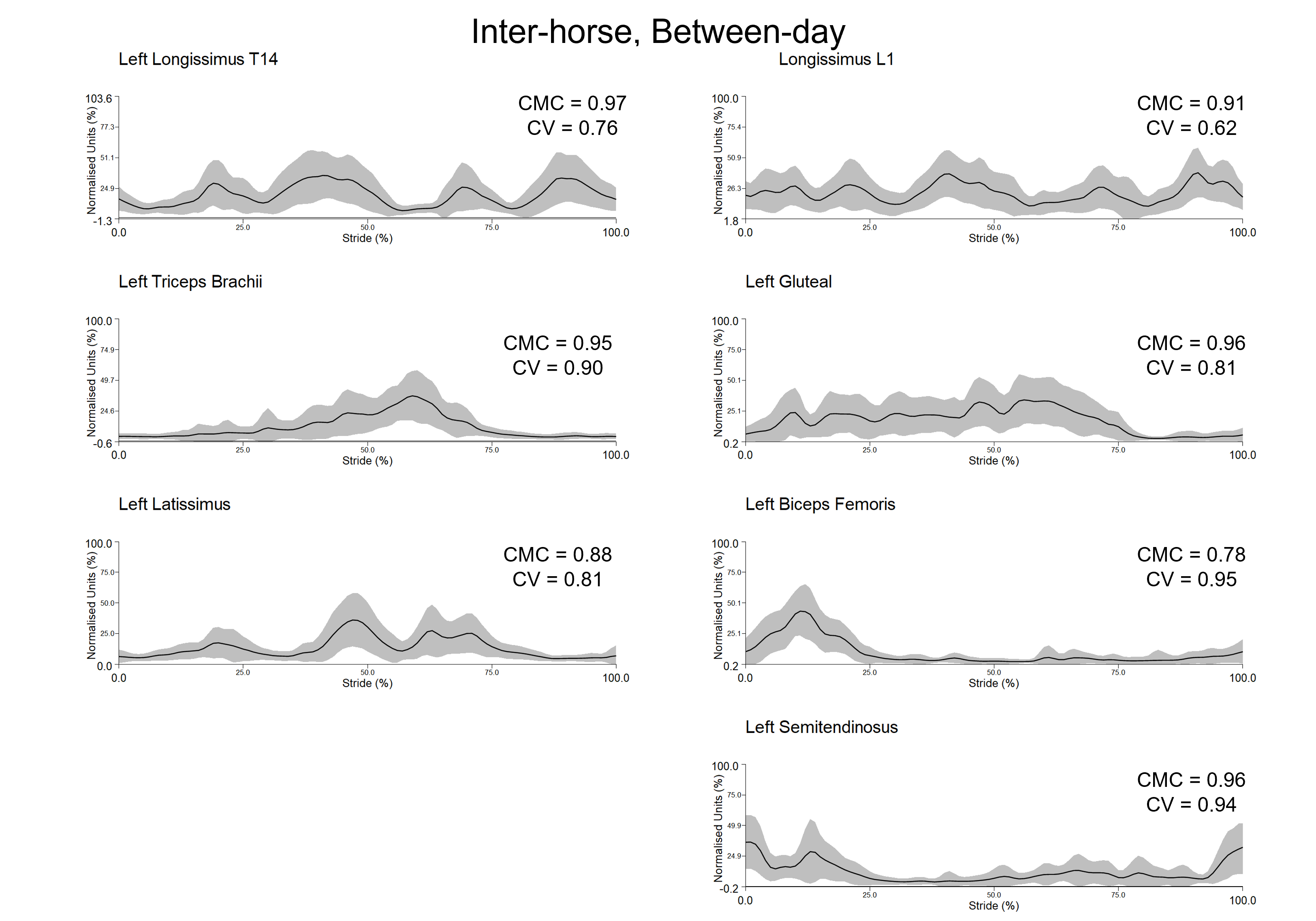

Supplement: S27 Fig — Mean (solid line) and standard deviation (grey shaded area) time and amplitude-normalised sEMG data from 152 trot strides are presented for each muscle. Coefficient of variation (CV) and coefficient of multiple correlation (CMC) is indicated for each muscle. (TIF) [file pone.0288664.s029.tif]
