# Supplementary material for: Reliability of surface electromyographic (sEMG) measures of equine axial and appendicular muscles during overground trot
Source: PLoS One. 2023 Jul 14;18(7):e0288664. doi: 10.1371/journal.pone.0288664 (PMC10348569; doi:10.1371/journal.pone.0288664)
Supplement: Attachment — Submitted filename: Response to Reviewers.docx [file pone.0288664.s030.docx]

We would like to thank the reviewers and editor for taking the time to review our manuscript and for providing such positive and constructive feedback. We have endeavoured to make all requested changes to the manuscript and feel that this has improved the quality of the work. All changes are highlighted as tracked changes within the resubmitted manuscript and we have responded to each of the reviewer/editor comments below in blue text. We would like to thank the reviewers and editor in advance for reviewing the resubmitted manuscript for publication within PLOS One.

**Reviewer #1:** This is a well-designed study. The introduction and discussion are very verbose and can be significantly shortened so that the reader does not get bogged down and miss the important points. Thank you for reviewing our manuscript and for your positive and constructive feedback.

Introduction:

The introduction is too long. It should be made more concise so that it covers the main points (briefly what is known in horses and humans, what the deficits are, justification of this study/gap in knowledge, objective/hypothesis). Much of the information that is more specific can be used in the discussion. Thank you, we have endeavoured to remove some information from this section to make it more concise. We do feel that much of the information provided is relevant for providing the reader with a sufficient background and justification for the study, so we were keen to maintain some of the detail from the original submission. We hope you will agree that our removal of some information is sufficient to mitigate your concerns.

Line 44: This sentence should have multiple references not just a single reference from a review article. We have now included additional references for this sentence.

Materials and Methods

Please include a paragraph on the study design. There seems to be 2 data collection session. When did these occur (same day, different day)? Please include this. Thank you for this suggestion. We have now included this information in lines 132 - 138.

Lines 128-132: Please delete these sentences. This can be referenced in the appropriate sections of the materials and methods. This has now been deleted from the manuscript.

Lines 135-136: What university herd (Utrecht, Central Lancashire?). Please add this information. Apologies for this omission. We have now specified that horses were from Utrecht University’s herd.

Line 136: How were horses managed during the study period? Stall, regular turn-out? How long was the study period? Was the study performed at one or both of the universities? Thank you for this suggestion. We have provided further detail on the management of the horses during the study period and the length of the study period in lines 143 - 147. We have clarified that the study was performed at Utrecht University in line 132 - 133.

Line 140: Add a sentence that gives a brief description of methods so that the reader does not have to go read the references. For example, placement of the markers and sEMG sensors is important. Since this is a repeatability study, how did you ensure accurate replacement of markers/sensors? Thank you for this suggestion. We have now included details on how we mitigated sensor re-application errors in lines 166 - 169. With respect, we would prefer to refer the reader to our previous studies which describe, in detail, sEMG sensor and retro-reflective marker locations. Repeating these locations in this study would substantially increase the length of the methods section and reviewers 2 and 3 have not requested this information, so we hope you will agree that it is sufficient to refer to our previous studies.

Line 181: Consider changing the section heading; data processing is a little more accurate (data analysis may be confused with statistical analysis). Thank you for this suggestion. The section heading has now been changed to “Data Processing”.

Line 184: Please include a little more information on how hindlimb impact was detected (what marker(s)). In addition to referencing the method by Roepstorff et al., we have stated that the maximum vertical displacement of the marker placed between the tubera sacrale was used for stride segmentation (line 200 - 201).

Lines 190-192: What joints were examined and was the back studied as a singular segment or multiples? Please include more information. We have now provided further information on the joint/segment angles in lines 205 - 212.

Line 225: What software was used to calculate these variables? Apologies for this omission. sEMG profiles were calculated in Visual3D and this has now been added to the manuscript.

Line 238: What summary statistics were calculated and presented? Thank you for noticing this omission. We have now specified that the mean ± standard deviation were calculated as summary statistics.

Lines 240-257: This paragraph is not appropriate for the statistical analysis section. This can be used in the discussion. The statistics section should present what statistics were performed not a discussion of why or what they mean. We agree and have now moved parts of this paragraph to the discussion section and have left only the relevant sentences that relate to the methods used.

Lines 266-273, 300-308: Instead of making a list of where all the data is contained, present pertinent data and then reference the tables/figures. Thank you for pointing this out. We have removed our description of the tables within these paragraphs and have instead referenced them where the associated data are described. However, we have not removed the sentences describing the figures and the data that these contain, as they present data from individual/representative horses, as well as from the group of horses (but only the right-side muscles in these instances). As such, these figures do not contain data from the entire dataset and therefore cannot be referenced in relation to the results that are presented in the text. We have shortened these sentences, so that they take up less space within the paragraphs but hope you will agree that it is helpful for the reader to have these descriptions of the Figures within the text.

Lines 334-335: The results section presents the results – does not interpret them. This sentence can be used in the discussion. Thank you for this suggestion. We have altered the sentence to remove the comparison with findings for within-session reliability. We hope you will agree that the revised sentence only presents the results.

Discussion

Lines 365-368: Please don’t include sentences about what you are about to discuss. This is unnecessary. Please delete these sentences. With respect, we would prefer to keep this sentence, as some readers may expect a discussion of sEMG activity profiles in relation to muscle function, particularly within the context of the joint/segment motion during trot. We feel it is important to state that our discussion will instead focus on the reliability of the sEMG profiles, and that the reader can refer to the “sister” manuscripts where the above information are described in detail. We hope you will agree with our reasons for including this sentence at the outset of the discussion section.

Lines 371-374: Please avoid writing sentences using “we”. You could state that these techniques have been previously used in humans and equine. Thank you, this has now been changed.

Lines 375-376: Please include a sentence or 2 about how your CVs compare to these reported ones. With respect, we would prefer not to make this comparison, as these CV thresholds have been used for discrete data only and are therefore not directly applicable to the continuous data that were used in our study. We explain this in the following sentence and make the suggested comparison with CV values that have been reported in other studies that employed comparable continuous data. As such, we hope you will agree that making such a comparison with the values in the highlighted lines would not be appropriate and may confuse the reader.

Lines 421-422: Do you think that investigation of muscle activation/timing may be a good variable to examine? This was identified as a variable that could change in response to lameness/underwater treadmill exercise (King et al AVJR 2017). Yes, we agree that this would be a relevant variable to study using the CV in future studies. We also noted that discrete onset/offset variables were significantly altered during induced fore- and hindlimb lameness in St. George et al. (2022) and Spoormakers et al. (2023). However, we felt that an examination of these variables went beyond the scope of this preliminary evaluation.

Line 436: Please change “is influenced” to “may be influenced”. This has now been changed.

Lines 452-458: Please delete these sentences. It is not necessary to describe this. Describe what others have identified and how this is related to what you found. We have now removed this introductory sentence.

Lines 458-465: This section can be made more concise by not repeating the results. It seems that you found differences in muscle groups between left and right sides. Has this been seen by other groups? Could this be from asymmetry, sidedness of the horse, difference in sensor placement? Thank you, we have now removed the results from these sentences and have included an additional sentence to touch on the bilateral differences in CMC that were observed for biceps femoris. We are not aware of other studies that have observed this in horses during trot.

Line 466: This seems to be a new thought process (not left vs right). A new paragraph could be started here. Thank you, we agree and have now created a new paragraph.

Lines 497-501: Please delete these sentences. Just state the limitations. The subheading is “limitations”. These sentences have now been deleted.

Lines 501-503: This sentence can be shortened. You have a small sample size that is not representative of all horses everywhere. This sentence has been revised.

Lines 560-578: Restate the conclusions. This can be done in a couple sentences. Don’t restate your hypotheses or your discussion. Do not include information on lameness as this is beyond the scope of this study. We have now made this section more concise and have removed the below sentence regarding lameness but would prefer to leave our mention of lameness in the concluding sentence, as this is included in the introduction as a reason for conducting this study and it highlights the potential impact of our findings.

Lines 573-575: Please delete this sentence. This sentence has been removed.

**Reviewer #2:** This is a well written paper with appropriate statistical analysis and minimal or no grammatical or spelling errors. The references no 13 and 14 are referred to in the materials and methods. They are not fully referenced as they are still online as at the time of your submission, but as these are required to be read so that the methods are understood, the full references for both these papers need to be included. Thank you for reviewing our manuscript and for your positive feedback. We have now updated reference 14 to include the volume and issue number, which have since been released since the initial submission of this manuscript. Unfortunately, Spoormakers et al. [13] is still published online as an “Early View” article (Online Version of Record before inclusion in an issue) in EVJ, so we cannot update the record further in this resubmission. We have, however, included the doi for this reference to ensure that it is accessible for the reader.

**Reviewer #3:** Summary: This manuscript aims to determine whether a typical profile of sEMG activity can be reliably described within- and between-horses for selected appendicular and axial superficial muscles during in-hand trot. As well as to determine the between-session reliability of these profiles. Increased knowledge of normative sEMG profiles could serve as a base for future aid in clinical decision-making. However, there is a general lack of knowledge about the reliability of equine sEMG profiles, so this manuscript is a useful addition to the literature. The study focus is on the within- and between horses reliability as well as the between- sessions reliability. Thank you for taking the time to review our manuscript and for providing such constructive feedback. We have endeavoured to make all requested within the manuscript and have addressed each of your comments below.

General comments

This is an interesting, overall very well written manuscript and useful contribution to the available literature regarding the potential use of sEMG in clinical decision-making. The design is well described and the results clearly laid out.

The paper could benefit from additional information regarding the clinical implications of the results, such as an elaboration of the cut-of values for CMS when choosing to use the method or not. If so, it would give potential users of the technique important information. Thank you for your constructive feedback and suggestions for improvement. Unfortunately, the preliminary nature of this study and the relatively small sample size means that we are not able to define or comment on CV or CMC cut-off values. We do agree that this is important information for using sEMG to evaluate muscle activity in horses and we have commented on the need for future studies to build on the results presented here, so that these cut-offs can be determined (lines 468 – 471, 590 – 592, 609 - 612). We hope you will agree that highlighting this requirement is sufficient for this preliminary study.

Abstract: This describes the study clearly. Thank you!

Introduction: The rationale for the study is well described and put into the clinical context. Thank you!

Methods: The methods are well described. Recommendations- to add the maximum period between sessions and to clarify if it was the same handler for all horses and all registrations. Thank you for these suggestions. We have now included the maximum period between sessions in lines 137 - 138 of the revised manuscript. In line 187, it is stated that one handler led the horses throughout the data collection sessions, but we have tried to clarify this.

Results: These are comprehensively reported. Recommendations- to clarify if the horses had any asymmetries before the induction of lameness. Thank you for the positive feedback on this section. We confirm in lines 143 – 144 and 190 – 194 that horses were deemed as clinically non-lame by two experienced equine veterinarians prior to, and during, the study. With respect, we would prefer not to report on asymmetries in the results section, as we are only reporting data from the baseline, or “non-lame”, condition and the potential impact of movement asymmetry on the results are described in detail in the discussion section (lines 539 - 545). We hope you will agree that this is the most appropriate way of addressing this.

Discussion: This is a reasonable discussion of the findings. However, it would be interesting to have some additional discussion regarding the selection of “Reference voluntary contraction” for other gaits than trot. Thank you for the positive feedback on this section. We would like to draw the reviewer’s attention to lines 569 - 581 within the limitations section, where we discuss the normalisation method used. The RVC employed for normalisation (the peak amplitude observed across trot strides within each horse, muscle, and test session) could be applied to sEMG signals, irrespective of the gait studied. With respect, we would prefer not to include this in our existing discussion of normalisation, as we feel it would disrupt the flow and may be confusing for some readers. We hope you will agree with this.

Conclusions: The conclusions are relevant and supported by the results. However, on line 570 it states that the study results provide “evidence for sEMG as a reliable measure of muscle activity”. Maybe one could add that the validity needs to be investigated in future studies. Thank you for this suggestion. We feel we’ve addressed the need for future validation studies in lines 609 - 612, where we state that “future research should build on this preliminary study to explore, and eventually validate, normative sEMG profiles for equine gait …”. We hope you will agree that this sentence addresses your suggestion for improving this section.

References: These are considered comprehensive. Thank you!

**Editorial comments:**

P2, l19. Is it possible to define the type of study? We have now included “observational” study within this sentence.

P3, l52.Is it possible to define what type of mechanisms? Apologies, we have now added “neuromuscular mechanisms” for clarity.

P5, l89. “groupaverage”- or group- average? We can confirm that this is correctly written as “group-averaged”.

P5, l96. 2932, should be 29, 32? We can confirm that this is referenced correctly as “29 – 32”.

P5, l97. “betweensession”- this is sometimes spelled as one word (with and without an s) and sometimes as between-session(s) in the manuscript (the same applies to some other words in the manuscript such as intra-subject, within-session(s) and for the abstract as well). Thank you for pointing this out. We hyphenate “between-session” when we refer to the form of reliability that we studied, but we do not hyphenate “between sessions” when we are describing the two separate data collection sessions that were conducted. However, we have endeavoured to make this more consistent throughout the manuscript as tracked changes.

P6, l118. Inhand- or in-hand? We can confirm that this is correctly written as “in-hand”

P10, l193. Commonmode- or common-mode? We can confirm that this is correctly written as “common-mode”

P10, l 220. Betweensessions- two words? We can confirm that this is correctly written as “between-session”

P12, l267. Intrasubject- one or two words? We can confirm that this is correctly written as “intra-subject”

P12, l273. Intrasubject- one or two words? We can confirm that this is correctly written as “intra-subject”

P14, l 306. Betweensessions- two words? We can confirm that this is correctly written as “between-session”

P19, l426. Betweenhorses- two words? Thank you for noticing this. We have now written this as two words (between horses).

P21, l467. Affects Thank you for noticing this, which we have now corrected.

P23, l5121 betweentest- two words? ? Thank you for noticing this. We have now written this as two words (between test).

P25, l565. Betweensession- two words? Thank you for noticing this. We have now written this as two words (between horses).
