# Supplementary material for: Reliability of surface electromyographic (sEMG) measures of equine axial and appendicular muscles during overground trot
Source: PLoS One. 2023 Jul 14;18(7):e0288664. doi: 10.1371/journal.pone.0288664 (PMC10348569; doi:10.1371/journal.pone.0288664)
Supplement: Attachment — Submitted filename: Response to Reviewers.docx [file pone.0288664.s031.docx]

We would like to thank the reviewers and editor for taking the time to review our manuscript in this second round and for providing such positive and constructive feedback. We have endeavoured to make all requested changes to the manuscript and feel that this has improved the quality of the work. All changes are highlighted as tracked changes within the resubmitted manuscript and we have responded to each of the reviewer/editor comments below in blue text. We would like to thank the reviewers and editor in advance for reviewing the resubmitted manuscript for publication within PLOS One.

**Reviewer #1:** The authors have done a nice job editing the manuscript based on previous recommendations. I have a few additional comments: Thank you for reviewing our manuscript and for your positive and constructive feedback.

Introduction:
While there is good information within the introduction, it is still too long. Because of this, the reader will miss the key details and be stuck in the weeds of the less relevant information. Thank you for providing suggestions for how we can further reduce the length of this section. We have endeavoured to make all requested changes and hope you will agree that it is now a more appropriate length that is suitable for publication.

In paragraph 1, lines 53-54 is not important background or justification for the study and can be deleted. This sentence has now been deleted.

Most of paragraph 2 is not relevant and can be condensed to several sentences. It seems that the main point of this paragraph is that expected variability of sEMG in normal horses needs to be determined. Lines 73-82 seems unnecessary. Thank you, lines 73 – 82 have now been deleted and we hope you will agree that this paragraph is now sufficiently condensed.

Specifics of human studies (such as the gaits that have been evaluated) are not necessary to make your point (lines 83-85); this sentence can be deleted since the relevant finding to your argument is in the next sentence. This sentence has now been deleted.

Lines 88-92: the first part of the sentence is good (you lose valuable information when you group average data). Please remove the second part of the sentence starting at “so it has been suggested…” Thank you, we have removed this portion of the sentence.

Lines 95-102: none of this adds to your argument. Please delete. This has now been deleted.

Lines 103-104: It is worthwhile to state that treadmill locomotion and over-ground locomotion are not the same. As most horses will not be evaluated when on a treadmill, it seems relevant to examine this overground. This would be a gap in knowledge that you would fill with this project. Thank you, we have now included a sentence in lines 118 – 120 to cover this point.

It is not necessary to have references for your hypothesis. Please remove “Based on previous sEMG literature from humans and equine subjects” (lines 109-110). Just state your hypothesis! Thank you, this has now been removed.

Materials and Methods

Line 143: What size clipper blade was used? Apologies for this omission, we have now included details on the clipper blade in line 163.

Line 145: Was the sensor placed over the middle/center of the muscle belly? Please include this information. Apologies for this omission, we have now clarified that the sensor was placed over the middle of the muscle belly in line 166.

Results:

Lines 247-248: I assume that this number of strides is for both forelimbs and hindlimbs. Yes, it is inclusive of all muscles studied (from the fore- and hindlimbs, and longissimus).

Lines 254-268: It is fine to reference the representative figures; however, present the pertinent results first and move the representative figures to the end of the paragraph. Start with line 260 (Across test sessions and muscles …) Thank you for this suggestion, we have now moved these sentences to the end of the paragraph.

Lines 287-297: Same as the comment above (present the results first followed by the representative images). Thank you, the suggested changes have been implemented, as above.

Discussion:
Lines 351-354: This sentence is not necessary. Please delete. This has now been deleted.

Lines 370-374: Instead of stating that there are no studies to report CVs in equine, you can just state that this is the first study to report these values in horses. Thank you, this has now been amended.

Lines 375-377: This sentence is a bit confusing. I don’t think you need the statement “across all muscles and test sessions. Thank you, we have now deleted this statement from the sentence.

Lines 377-379: Was this data from the same data collection session as the current study? If so, this should be deleted. Yes, it was, so we have now deleted this sentence.

Lines 388-392: This is a very long and confusing sentence. This sentence could be separated into 2 which may help. There seem to be multiple trains of thought in this sentence (force impulse and electrode-skin interface). Please revise. Thank you, we have now split this into two sentences for clarity.

Lines 394-397: These 2 sentences state the same thing. Delete the first sentence (Small mean values…). Thank you, we have now deleted the first sentence.

Lines 426-427: Do you mean that you did not see the same magnitude of disparity? Please clarify. Apologies, we intended to state that our overall finding of higher CVs for inter- vs. intra-subject sEMG profiles agrees with the human literature. We have amended this sentence for clarity.

Line 457: Please replace “is” with “was” Thank you for noticing this error, which we have now corrected.

**Reviewer #3:** Thank you for revising the manuscript and in for some comments, answering why you did not. I have no further comments. Thank you for reviewing our revised manuscript and for your positive and constructive feedback.
